# Inducible Deletion of YAP and TAZ in Adult Mouse Smooth Muscle Causes Rapid and Lethal Colonic Pseudo-Obstruction

**DOI:** 10.1016/j.jcmgh.2020.09.014

**Published:** 2020-09-28

**Authors:** Fatima Daoud, Johan Holmberg, Azra Alajbegovic, Mario Grossi, Catarina Rippe, Karl Swärd, Sebastian Albinsson

**Affiliations:** Department of Experimental Medical Science, Lund University, Lund, Sweden

**Keywords:** WWTR1, YAP1, Gastrointestinal, Contractility, TEAD, Constipation, Muscarinic, CCh, carbachol, Ctrl, control, DAPI, 4′,6-diamidino-2-phenylindole, GI, gastrointestinal, HRP, horseradish peroxidase, mRNA, messenger RNA, MRTF, myocardin-related transcription factor, mT/mG, membrane-targeted tandem dimer tomato/ membrane-targeted green fluorescent protein, *P*adj, adjusted *P* value, SRF, serum response factor, TEAD, TEA domain, Y/T KO, YAP/TAZ knockout

## Abstract

**Background & Aims:**

YAP (*Yap1*) and TAZ (*Wwtr1*) are transcriptional co-activators and downstream effectors of the Hippo pathway, which play crucial roles in organ size control and cancer pathogenesis. Genetic deletion of YAP/TAZ has shown their critical importance for embryonic development of the heart, vasculature, and gastrointestinal mesenchyme. The aim of this study was to determine the functional role of YAP/TAZ in adult smooth muscle cells in vivo.

**Methods:**

Because YAP and TAZ are mutually redundant, we used YAP/TAZ double-floxed mice crossed with mice that express tamoxifen-inducible CreER^T2^ recombinase driven by the smooth muscle–specific myosin heavy chain promoter.

**Results:**

Double-knockout of YAP/TAZ in adult smooth muscle causes lethality within 2 weeks, mainly owing to colonic pseudo-obstruction, characterized by severe distension and fecal impaction. RNA sequencing in colon and urinary bladder showed that smooth muscle markers and muscarinic receptors were down-regulated in the YAP/TAZ knockout. The same transcripts also correlated with YAP/TAZ in the human colon. Myograph experiments showed reduced contractility to depolarization by potassium chloride and a nearly abolished muscarinic contraction and spontaneous activity in colon rings of YAP/TAZ knockout.

**Conclusions:**

YAP and TAZ in smooth muscle are guardians of colonic contractility and control expression of contractile proteins and muscarinic receptors. The knockout model has features of human chronic intestinal pseudo-obstruction and may be useful for studying this disease.

## Graphical abstract

**Figure:**
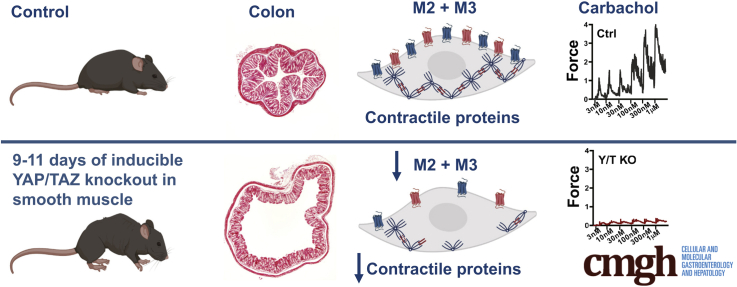

**Summary**

In this article, we examine the role of YAP/TAZ in gastrointestinal smooth muscle using an inducible knockout model. Deletion of YAP/TAZ caused rapid lethality resulting from colonic pseudo-obstruction. This was associated with reduced expression of muscarinic receptors and contractile proteins.

YAP (*Yap1*) and TAZ (*Wwtr1*) are transcriptional co-activators inhibited by large tumor suppressor kinase in the Hippo signaling pathway.^1^, ^2^, ^3^ YAP and TAZ play essential roles in organ size control via effects on cell proliferation and survival,^1^^,^^2^ and they mediate many of their biological functions via TEA-domain (TEAD) transcription factors.^4^^,^^5^ YAP and TAZ can be activated by substrate stiffness, actin dynamics,^6^ and via G-protein–coupled receptors independently of the Hippo pathway.^7^ YAP and TAZ are inhibited by cell–cell contact, allowing for cell density–dependent regulation of proliferation.^8^ Because of their importance in growth control, YAP and TAZ have attracted considerable attention in the cancer field, and efforts are made to target them for cancer therapy, including cancers arising in the gastrointestinal (GI) tract.^3^^,^^9^^,^^10^

Another family of transcriptional co-activators that is controlled by mechanical cues and actin dynamics similar to YAP/TAZ is the myocardin-related transcription factors (MRTF-A, MRTF-B, and myocardin).^11^, ^12^, ^13^ MRTFs activate transcription of their target genes through interaction with serum response factor (SRF). In smooth muscle cells, myocardin and MRTF-B are crucial in promoting the differentiated state of smooth muscle cells during development, and when their drive is lost smooth muscle cells lose their contractile properties to become proliferative and synthetic, a process referred as *phenotypic modulation*.^14^, ^15^, ^16^ As a result, constitutive knockout of myocardin leads to early embryonic lethality with halted development of smooth muscle cells in the aorta.^17^ In adult mice, inducible knockout of myocardin leads to a remarkable dilation of the intestine, bladder, and aorta, attributable to a loss of contractility in these tissues.^18^ As a result, these mice develop aortic aneurysms and aortic dissection and die from defects in vascular and visceral tissues within 6 months of myocardin gene deletion. Although the co-activators of SRF clearly are important for smooth muscle survival and function, the full role of SRF is shown in inducible smooth muscle–specific SRF knockout mice that die within 4 weeks after induction owing to severe GI pseudo-obstruction with impaired motility.^19^, ^20^, ^21^ MRTFs share certain target genes with YAP/TAZ,^22^ and recent studies have shown that these pathways may be interdependent in part owing to direct physical interaction,^23^^,^^24^ but also owing to downstream effects on actin dynamics.^25^

Cell-specific and inducible transgenic strategies have shown that YAP/TAZ control the development of arteries^26^, ^27^, ^28^ and GI mesenchyme,^29^ but no such strategies have addressed the role of YAP and TAZ in adult smooth muscle. This is warranted in view of the emerging notion that YAP and TAZ play important roles in mature smooth muscle. For example, it was argued that YAP and TAZ promote phenotypic modulation of arterial smooth muscle cells toward the synthetic phenotype to cause neointima formation.^30^^,^^31^ The latter findings are difficult to reconcile with the demonstrated mutual dependence of YAP/TAZ and MRTFs on each other. Most likely, the net impact of YAP and TAZ in smooth muscle can be gauged only by targeted and inducible deletion in vivo.

Our own interest in YAP and TAZ stems from correlation analyses at the messenger RNA (mRNA) level to identify novel target genes of myocardin.^22^^,^^32^ It was noted that YAP/TAZ and TEADs correlate tightly with myocardin across human tissues, especially in the GI tract and arteries. This motivated us to generate an inducible and smooth muscle–specific knockout of YAP and TAZ that we describe here, and that rapidly succumbs to colonic pseudo-obstruction owing to reduced expression of contractile proteins and muscarinic receptors.

## Results

### YAP/TAZ KO in Smooth Muscle of Adult Mice Results in Lethality Within 2 Weeks

**Figure 1:**
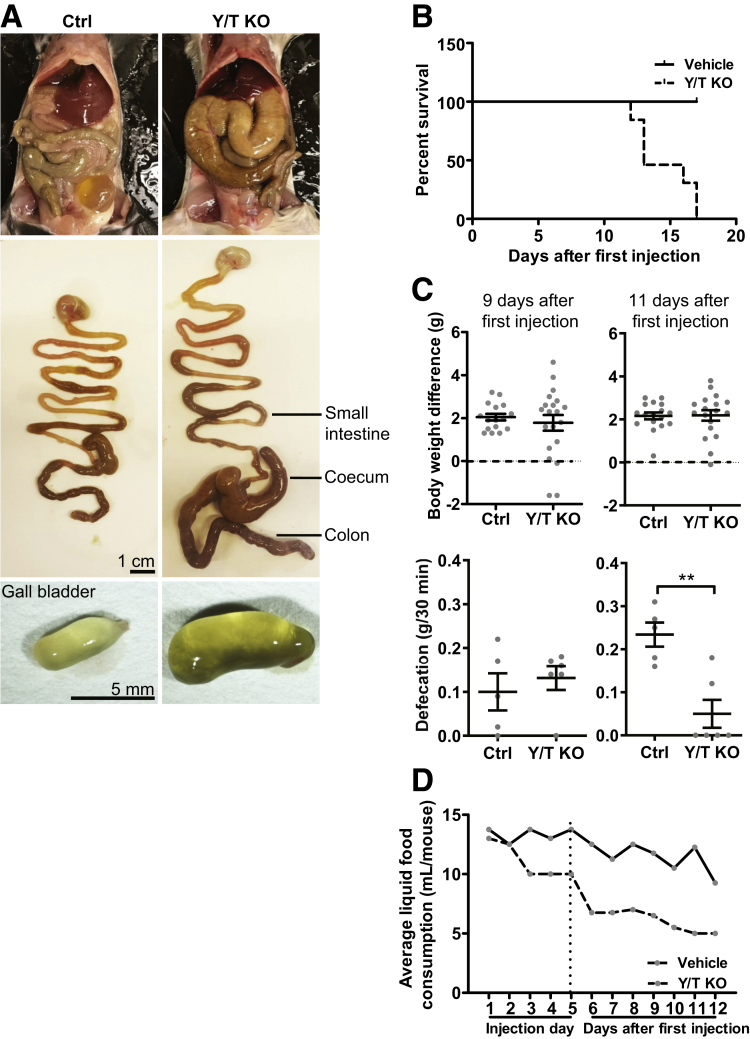
**Inducible and smooth muscle–specific Y/T KO is lethal, causing colonic swelling, reduced defecation, and reduced intake of liquid diet**. Five- to 8-week-old mice were killed 2 weeks after the first injection of tamoxifen. (*A*) *Top*: Opening the abdomen showed that the cecum and colon were swollen. *Middle*: This also was evident when the stomach and intestines were removed and photographed. *Bottom*: The only other organ that consistently was distended was the gallbladder, and internal thrombi were occasionally seen. (*B*) In another cohort with 6 vehicle-treated controls and 6 tamoxifen-treated Y/T KO mice, all KO mice died within 17 days of the first tamoxifen injection. (*C*) Body weights were indistinguishable at 9 (*left*) and 11 (*right*) days (*top*, *n* ≥ 16), but defecation was reduced at 11 days (*bottom*; *n* ≥ 5). In an attempt to reduce suffering, mice were fed a liquid diet, and the volume of liquid diet was measured on a daily basis (average of food intake per cage in 1 cage with 4 vehicle-treated controls and 1 cage with 4 tamoxifen-treated Y/T KO mice). (*D*) Consumption of a liquid diet started to decrease on the day of the third injection in the KO cage, and remained lower until day 12 when mice were killed. ∗∗*P* < .01 compared with control (Ctrl).

YAP (*Yap1*) and TAZ (*Wwtr1*) are transcriptional co-activators downstream of the Hippo signaling pathway. Recent studies have shown roles of YAP and TAZ in development of the gastrointestinal mesenchyme,^29^ and in coronary vascularization.^27^ YAP and TAZ also have garnered considerable attention because they are mechano-activated, and regulated by actin dynamics,^6^ suggesting that they may play important homeostatic roles beyond development in smooth muscle cells of adult mice.^30^^,^^31^^,^^33^^,^^34^ To explore the hypothesis that YAP and TAZ play important roles in mature smooth muscle, we crossed mice with floxed YAP and TAZ loci^18^ with mice that express tamoxifen-inducible Cre-recombinase under the control of a smooth muscle–specific promoter (smooth muscle myosin heavy chain, *Myh11*^35^). For knockout induction, tamoxifen was injected in the abdomen of 5- to 8-week-old male mice for 5 consecutive days (Y/T KO). Cre-negative floxed mice of the same sex were treated with tamoxifen in parallel and used as controls (Ctrl). The first cohort of Y/T KO was planned to be killed 4 weeks after injection, but the termination criteria stipulated in the ethical permit (reduced mobility, kyphosis, or ruffled fur) were met after 2 weeks and 2 mice already had died. The remaining mice in that cohort therefore were killed. Pathologic examination showed that the cecum and colon were grossly distended with hardened fecal contents (Figure 1*A*). The gallbladder was enlarged (Figure 1*A*), and in some cases discolored from bleeding. No apparent phenotype was observed in more proximal parts of the GI tract. In a second cohort of mice, the ethical end point criteria were met for all KO mice between days 12 and 17 (Figure 1*B*). Single KO or heterozygous deletions, such as YAP^-/-^/TAZ^+/+^ or YAP^+/-^/TAZ^-/-^, did not cause any gross phenotype in the GI tract. Taken together, this shows that smooth muscle YAP and TAZ are mutually redundant and that together they are critical for survival in adult mice, owing to an essential role in the distal GI tract.

To explore pathologic events preceding death, mice were examined on days 9 and 11 after the first injection. The body weights at these time points were indistinguishable between genotypes (Figure 1*C*). When mice were kept in individual cages to collect the fecal excrement for weighing, we found a reduced fecal output on day 11 (Figure 1*C*). In an attempt to prolong life and reduce suffering, mice were fed a liquid diet from the first injection onward. This allowed us to record liquid food intake, which started to decrease by the third day of tamoxifen injection and then continued to decrease until day 12 when the mice had to be killed (Figure 1*D*). Because termination criteria were met on days 12–17 irrespective of diet, the liquid diet strategy was abandoned, and mice henceforth were maintained on regular chow and killed on day 11.

### Colonic Remodeling in YAP/TAZ KO Mice

**Figure 2:**
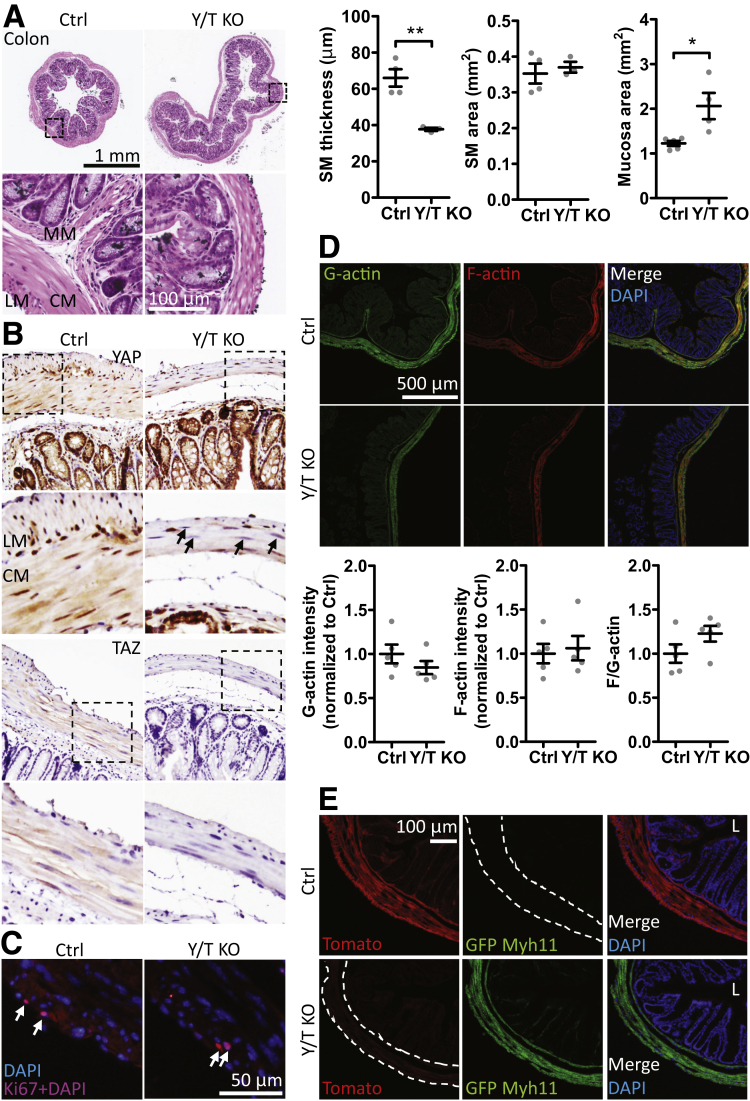
**Colonic distension with thinning of longitudinal and circular smooth muscle layers, and reduced expression of YAP and TAZ in Y/T KO compared with control mice**. (*A*) Smooth muscle (SM) (longitudinal and circular muscle layer) thickness, area, and mucosal area were measured and quantified on paraffin cross-sections of colon stained with H&E (*n* ≥ 3). (*B*) Immunohistochemical staining for YAP and TAZ. Nuclei of the SM layer showed reduced YAP staining (*arrows*). (*C*) Staining of the cryosectioned colonic SM layer with proliferation marker Ki67. (*D*) Confocal images and quantification of colonic SM G-actin (DNaseI) and F-actin (phalloidin) (*n* ≥ 5). (*E*) Cre-driven recombination switched the ROSA^mT/mG^ fluorescence from red to green in the outer muscle layers and the muscularis mucosa of the colon. No green fluorescence was observed in the epithelium. *Dashed lines* outline the SM layer. Nuclei were stained with DAPI. ∗*P* < .05 and ∗∗*P* < .01 compared with control (Ctrl). Five- to 8-week-old mice were used. CM, circular muscle layer; GFP, green fluorescent protein; L, lumen; LM, longitudinal muscle layer; MM, muscularis mucosa.

The longitudinal and circular smooth muscle layers were thinner in Y/T KO mice compared with controls (Figure 2*A*). The cross-sectional area of the smooth muscle layers, however, was unchanged, suggesting colonic remodeling. The area of the mucosa on the other hand appeared enlarged (Figure 2*A*). Immunohistochemistry supported nuclear and some cytoplasmic localization of YAP in longitudinal and circular smooth muscle cells of control mice, and this staining was reduced dramatically or lost in the muscular layers of Y/T KO mice (Figure 2*B*). Staining for TAZ was primarily cytoplasmic in smooth muscle cells of control mice, and this staining was lost in the Y/T KO mice (Figure 2*B*). Considering the role of YAP and TAZ in cell proliferation, we stained colon sections for Ki67 but no difference in cell proliferation was observed in the Y/T KO smooth muscle layer compared with control (Figure 2*C*) (mean % Ki67-positive smooth muscle cells ± SEM: Ctrl, 0.53 ± 0.108; KO, 1.33 ± 0.438; *P* = .23). Moreover, no change in smooth muscle cell number was observed as measured by nuclear staining 4′,6-diamidino-2-phenylindole (DAPI), supporting that there is no loss of smooth muscle cells in Y/T KO colon (mean number of nuclei/μm^2^ ± SEM: Ctrl, 0.34 ± 0.002; KO, 0.37 ± 0.003; *P* = .49). A previous report from Mericskay et al^20^ described reduced levels of filamentous actin (F-actin) in colonic smooth muscle upon inactivation of the SRF gene. No change in F-actin, G-actin, or the F-actin:G-actin ratio was observed in the Y/T KO colon (Figure 2*D*). In view of the expansion of the mucosal area, we used a dual-color fluorescent Cre-reporter model (ROSA^mT/mG^) to examine Cre-driven recombination events. Cre-negative mice expressed membrane-bound tandem dimer tomato (mT) with red fluorescence in the longitudinal and circular muscle layers, muscularis mucosa, and, more faintly, in epithelial cells (Figure 2*E*). After tamoxifen treatment of Cre-positive mice, the red fluorescence in the smooth muscle cells was replaced by green fluorescence of membrane-bound green fluorescent protein (mG) indicating Cre-mediated recombination. No green fluorescence was seen in the mucosa as shown using co-staining with DAPI to visualize nuclei (Figure 2*E*, bottom right). Taken together, these findings corroborate colonic remodeling and resultant thinning of the smooth muscle layer, and rules out that expansion of the mucosal area is the result of partial loss of YAP and TAZ in the mucosa.

### YAP/TAZ KO Causes Reduced Expression of Genes Important for Colonic Motility

**Figure 3:**
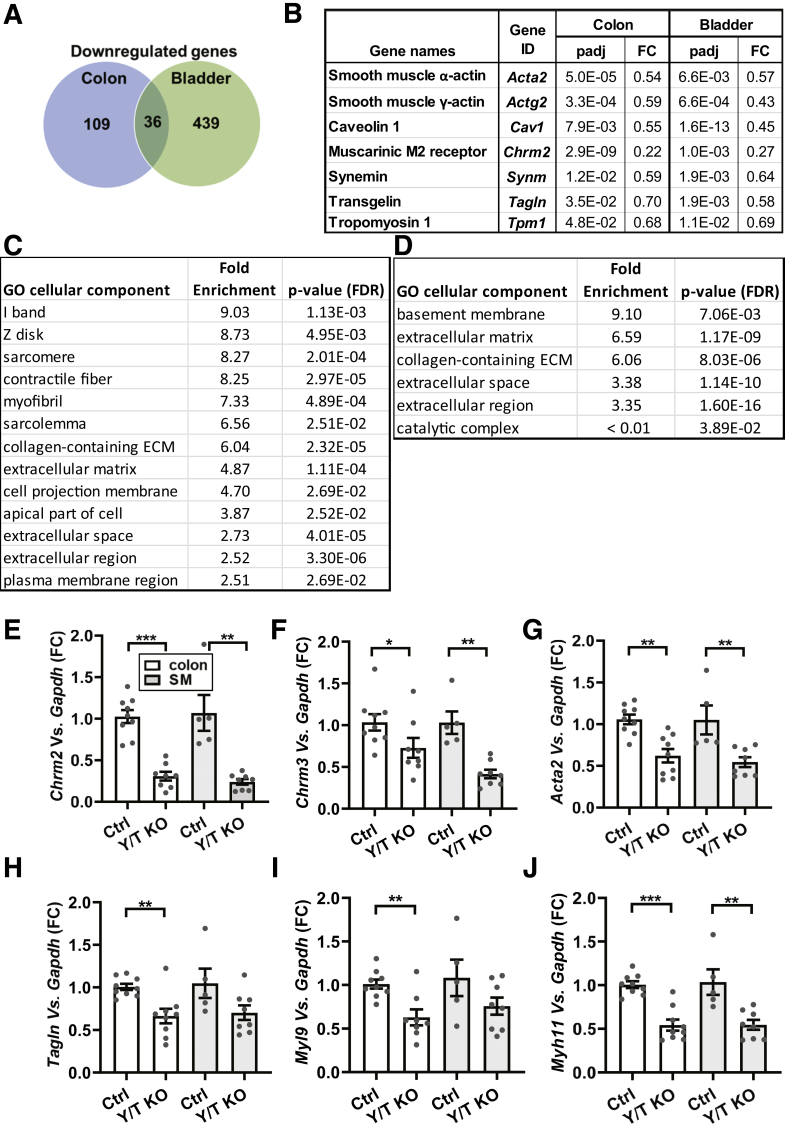
**Reduced expression of genes involved in contractility in the colon and urinary bladder of Y/T KO mice**. RNA was isolated from the colon and urinary bladder of 5- to 8-week-old mice and subjected to RNA sequencing. (*A*) Venn diagram shows the down-regulated genes. (*B*) Selected examples of transcripts from the overlay and their corresponding fold change and *P*adj values are represented. Gene ontology enrichment analysis was performed by the PANTHER Overrepresentation Test using the gene ontology database for either (*C*) down-regulated or (*D*) up-regulated genes in the Y/T KO colon. The Fisher exact test and correction for false discovery rate (FDR) were used for statistical analysis. n = 3. (*E–J*) Reverse-transcription quantitative polymerase chain reaction data of selected transcripts from the commonly down-regulated genes (*n* ≥ 5). Whole-colon samples (*white bars*) and samples where the outer muscle layers were isolated by microdissection were compared (*gray bars*). ∗*P* < .05, ∗∗*P* < .01, and ∗∗∗*P* < .001. ECM, extracellular matrix; FC, fold change.

To gain molecular insight into the colonic phenotype, RNA sequencing was performed on bladder and colon tissues from Y/T KO and control mice. The urinary bladder appeared grossly normal in Y/T KO and control mice. Several hundred genes were expressed differentially, and many were unique for the colon and bladder (Figure 3*A* and Supplementary Table 1). A Gene ontology enrichment analysis was performed on significantly up-regulated and down-regulated genes in the colon. The results from this analysis indicated a significant enrichment of genes associated with contractile function and the extracellular matrix (Figure 3*C* and *D*). Among the uniquely up-regulated transcripts in the colon were *Saa1* (37840-fold, *P* adjusted [*P*adj] = 3.2 × 10^-99^) and *Saa2* (203-fold, *P*adj = 2.4 × 10^-6^), which are acute-phase proteins known to be induced in GI diseases. In search of underlying mechanisms, we focused on down-regulated genes that were shared between the colon and the bladder, reasoning that these are most likely target genes of YAP and TAZ. Thirty-six transcripts were found in this overlay (Figure 3*A*), including several contractile smooth muscle markers such as *Tpm1*, *Actg2*, *Acta2*, *Cav1*, *Synm*, and *Tagln* (Figure 3*B*). Interestingly, we observed that the muscarinic M_2_ receptor (*Chrm2*) was repressed in both tissues (fold change in KO vs Ctrl colon, 0.22; *P*adj = 2.95 × 10^-9^; bladder, 0.27; *P*adj = .001). Furthermore, the muscarinic M_3_ receptor (*Chrm3*) was reduced in bladder (fold change in KO vs Ctrl bladder, 0.41; *P*adj = 8.82 × 10^-7^), while the change did not reach statistical significance using the adjusted *P* value in the colon (fold change in KO vs Ctrl colon, 0.33; *P*adj = .15). This suggested that colonic motility may be impaired owing to reduced levels of muscarinic receptors and contractile proteins.

We next sought to confirm a selection of the mRNA changes using reverse-transcription quantitative polymerase chain reaction. For this we compared the whole colon, including mucosa, with samples in which the outer smooth muscle layers were isolated by dissection. The muscarinic receptors *Chrm2* and *Chrm3* showed the same relative reduction in the whole colon samples as in the RNA sequencing experiment (Figure 3*E* and *F*). Importantly, both *Chrm2* and *Chrm3* were reduced significantly in Y/T KO compared with Ctrl using isolated colonic smooth muscle. The opposite pattern was seen for some of the contractile markers. *Myl9* and *Tagln*, for example, were reduced in whole colon, but were not reduced significantly in the isolated muscle (Figure 3*H* and *I*). Two of the contractile markers, *Myh11* and *Acta2*, were reduced both in whole colon and in isolated muscle (Figure 3*G* and *J*).

**Figure 4:**
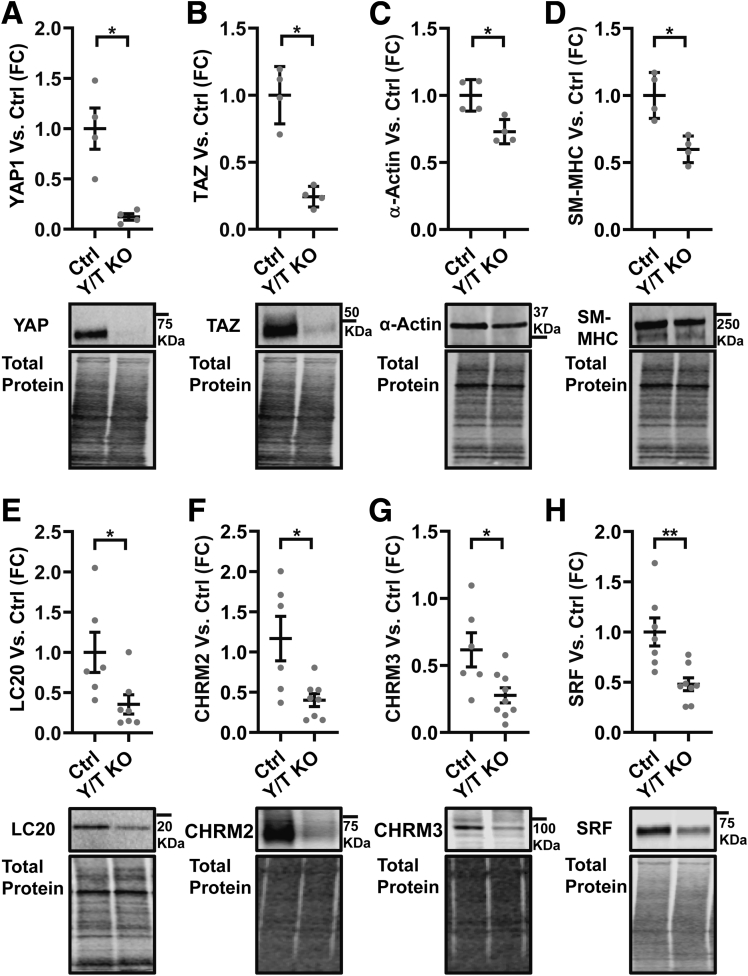
**Reduced protein expression of SRF, contractile markers, and muscarinic receptors in the Y/T KO colon**. (*A–H*) Representative Western blots using specific antibodies for YAP, TAZ, SRF, selected contractile proteins (α-actin, myosin heavy chain [SM-MHC], myosin light chain [LC_20_]) and muscarinic receptors (CHRM2 and CHRM3) (*n* ≥ 4). Western blot data were quantified by normalizing to total protein stain of the same lane. ∗*P* < .05 and ∗∗*P* < .01. Five- to 8-week-old mice were used. FC, fold change.

Protein expression next was examined to corroborate reduction of contractile proteins and muscarinic receptors. Western blot showed that YAP and TAZ were reduced in Y/T KO as expected (Figure 4*A* and *B*), as were smooth muscle α-actin, smooth muscle myosin heavy chain, and myosin light chain (Figure 4*C–E*). Western blot for muscarinic receptors resulted in several bands. However, major protein bands at the expected molecular weights (60–70 kilodaltons for M_2_ and 80–90 kilodaltons for M_3_) were down-regulated consistently in Y/T KO colon (Figure 4*F* and *G*).

A mutual dependence of MRTF–SRF and the YAP–TEAD pathways have been reported.^25^ We accordingly found that SRF was reduced significantly in Y/T KO colon (Figure 4*H*) whereas MRTF-A expression was unchanged (means ± SEM: Ctrl, 1 ± 0.144; KO, 0.78 ± 0.155; *P* = .32). Several proteins with critical roles in GI contractility thus were reduced in the Y/T KO colon along with 2 important muscarinic receptors.

### Expression of YAP and TAZ Correlates With Muscarinic Receptors and Regulators of Smooth Muscle Contractility in Human GI Tissues

**Figure 5:**
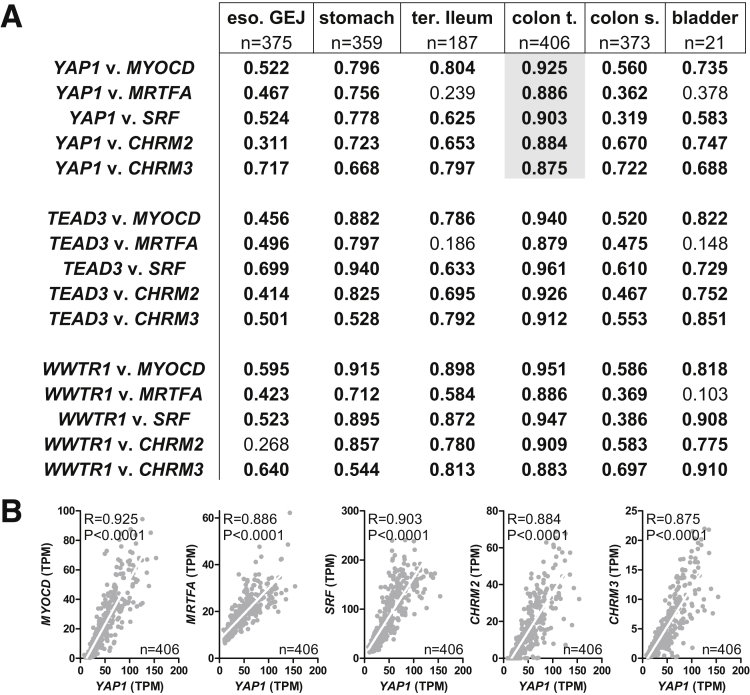
**Correlation between YAP/TAZ, MRTF/SRF, and muscarinic receptors in the human GI tract and urinary bladder**. (*A*) For the table, RNA sequencing data from GTExPortal.org was used to correlate mRNA levels of *YAP1*, *TEAD3*, and *WWTR1* vs *MYOCD*, *MRTFA*, *SRF*, *CHRM2*, and *CHRM3*. Spearman Rho values for the indicated correlations are given for 5 sections of the GI tract and the urinary bladder. All Spearman Rho values in bold have a *P* value less than .0001. Examples of correlations in the transverse colon are highlighted in gray. (*B*) Examples of correlations in the transverse colon are plotted. *White lines* are best-fit linear regression lines. eso GEJ, esophagus gastroesophageal junction; colon s., colon sigmoid; colon t., colon transverse; ter. ileum, terminal ileum; TPM, transcripts per million.

Our data indicate that YAP and TAZ are important for expression of contractile proteins, possibly through an effect on myocardin/SRF, and for muscarinic receptors. This should be reflected in correlations at the mRNA level, and we accordingly examined this using human expression data (RNA sequencing data from GTExPortal.org) with the aim to increase the translational impact of our findings. *YAP1*, *TEAD3*, and *WWTR1* all were found to correlate tightly with *MYOCD*, *MRTFA*, *SRF*, *CHRM2*, and *CHRM3* throughout the human GI tract and in the urinary bladder (Figure 5*A*). Correlations were particularly impressive in the transverse colon, and 5 examples are shown in Figure 5*B*.

### Impaired Colonic Contractility in YAP/TAZ KO Mice

**Figure 6:**
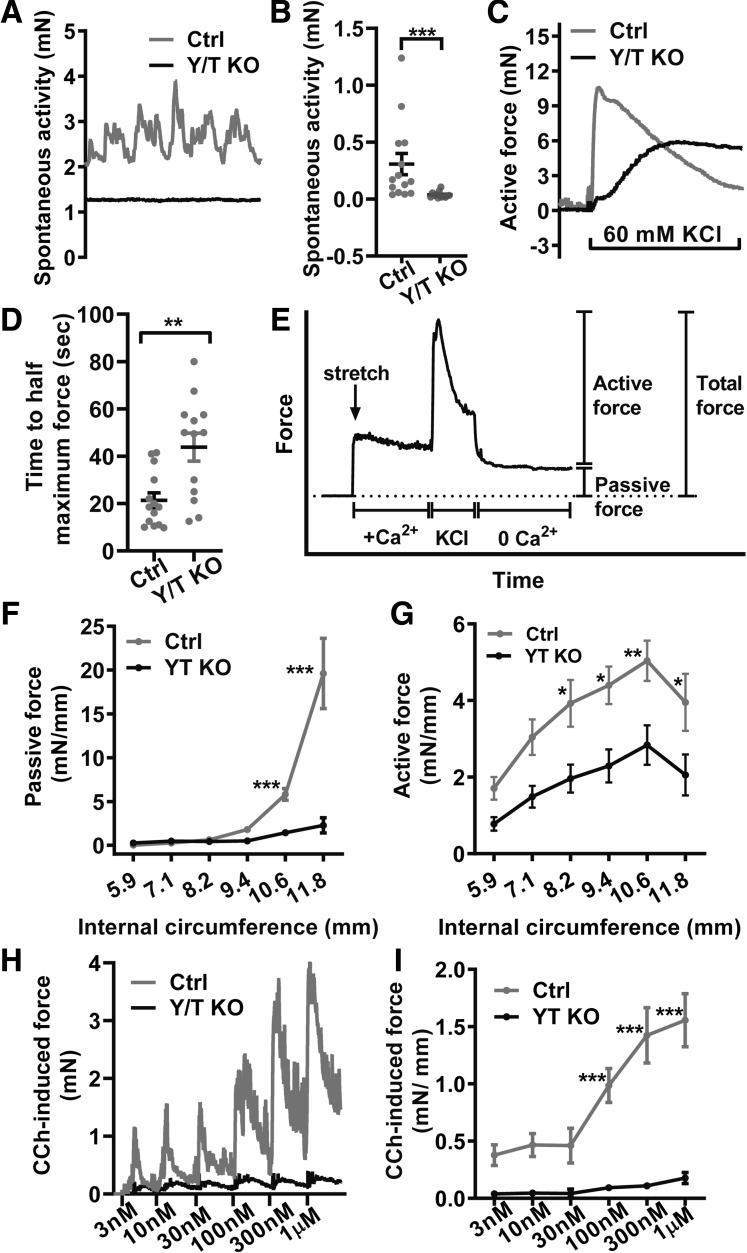
**Generalized impairment of colonic contractility in Y/T KO mice**. Colonic rings (3-mm long) of 5- to 8-week-old mice were cut and mounted on pins in multichannel myographs to measure force. Spontaneous contractile activity developed in essentially all control preparations (*gray line*), but rarely in Y/T KO preparations (*black line*) as shown by the (*A*) original traces and the (*B*) summarized data calculated as integrated force for 5 minutes. (*C*) Contraction in response to membrane depolarization was delayed in the Y/T KO colon, with a severe reduction of the early peak, and a much smaller effect on sustained force. (*D*) Accordingly, the half time of depolarization-induced contraction was much longer in the Y/T KO preparations. (*E*) Length–tension relationship protocol in which colon rings were stretched to specific internal circumferences and equilibrated in Ca^2+^-HEPES. Subsequently, 60 mmol/L KCl was added for 7 minutes and colon rings were allowed to relax in Ca^2+^-free HEPES at the end. (*F*) Total force was calculated by integrating force throughout the stimulation period with KCl, while the passive force (*n* ≥ 5) was determined by integration over 10 minutes in Ca^2+^-free solution. (*G*) Active force (*n* ≥ 5) is the result of subtracting the passive force from the total force. (*H*) Force traces on stimulation with the muscarinic receptor agonist CCh. (*I*) Complete concentration-response relationships for CCh calculated as the integrated force over 2 minutes of each concentration (*n* ≥ 4). ∗*P* < .05, ∗∗*P* < .01, and ∗∗∗*P* < .001.

To investigate the functional importance of altered gene expression in Y/T KO smooth muscle, colonic preparations were mounted in myographs for force measurements. At the same basal tension, spontaneous contractile activity developed in essentially all control preparations, but was absent in most preparations from Y/T KO mice (Figure 6*A* and *B*). Contractions elicited by membrane depolarization (60 mmol/L K^+^) also were very different, developing slowly with an increase time to half maximum contraction, but with a sustained phase that was at least as large as that in controls (Figure 6*C* and *D*). To better understand if remodeling affected the optimal circumference for force development, we systematically varied the internal circumference and measured active force in response to depolarization and passive force in the absence of Ca^2+^ at each circumference (Figure 6*E*). Passive force was reduced considerably at circumferences greater than 10 mm (Figure 6*F*), whereas active force was reduced significantly at circumferences greater than 8 mm (Figure 6*G*; measured here by subtracting passive force from the integral force throughout the KCl stimulus period). We finally generated concentration-response relationships for the muscarinic agonist carbachol (CCh). The response to CCh was severely reduced across the entire concentration range (Figure 6*H* and *I*). Taken together, these studies show that YAP and TAZ knockout in adult mice impairs colonic contractility, especially that elicited by muscarinic-receptor stimulation, causing colonic pseudo-obstruction and lethality.

## Discussion

Herein, we tested the novel concept that YAP and TAZ play roles in colonic homeostasis via smooth muscle autonomous effects in adult mice. Inducible Cre-driven deletion of YAP and TAZ in smooth muscle reduced levels of the M_2_ and M_3_ receptors and contractility, causing colonic pseudo-obstruction and death within 17 days. Colonic motility is impaired in M_2_/M_3_ knockout mice and defecation is reduced.^36^, ^37^, ^38^ However, M_2_/M_3_ knockout mice do not die spontaneously, and we found that the reduced muscarinic contractility in Y/T KO mice was compounded by a generalized reduction of force-generating capacity, resulting, at least in part, from repression of contractile proteins such as α-actin and myosin heavy chain. In the latter regard, the phenotype of Y/T KO mice is similar to inducible and smooth muscle–specific deletion of SRF in adult mice. Adult SRF mutants die within 16–28 days, with distension of the cecum and colon, reduced GI transit, and reduced muscarinic contraction.^19^, ^20^, ^21^ This is caused primarily by reduced levels of contractile proteins. Interestingly, expression of MRTF-SRF targets is dependent on YAP-TEAD activity,^25^ and our results suggests the SRF expression is reduced in Y/T KO colon. This provides a likely explanation for the reduced expression of contractile proteins and contractile function. Taken together, the results suggest that the severe phenotype of the Y/T KO mouse model arises owing to compound effects on the receptor level and on the contractile apparatus of smooth muscle cells.

A rapid lethal phenotype caused by colonic pseudo-obstruction does not occur in various heterozygous combinations of YAP and TAZ, but requires inactivation of all 4 alleles. Changes in contractility may follow a different pattern, but this has yet to be determined. The novel insights presented in this article may guide clinical studies of human pseudo-obstruction, which is a disabling form of GI dysmotility for which YAP and TAZ have not been implicated previously. Although the expression of YAP/TAZ in human disease was not investigated herein, we report highly significant positive correlations of YAP/TAZ/TEAD with muscarinic receptors and regulators of smooth muscle–specific gene expression throughout the human GI tract. This is consistent with an upstream regulatory role of YAP and TAZ for expression of these transcripts in human beings as well.

YAP/TAZ are likely to be deleted from smooth muscle cells in all tissues in the Y/T KO, but in the short time frame used in this study we observed an apparent phenotype only in the colon, cecum, and gallbladder, which all were distended severely at 9–11 days after the first tamoxifen injection. Other smooth muscle–containing tissues such as the stomach, small intestine, and urinary bladder appeared normal, but we did not perform a physiological analysis in these tissues. It is likely that Y/T KO smooth muscle cells in other parts of the GI tract show reduced contractile function but that propulsion of fecal contents requires an increase of force generation by GI smooth muscle because the hydration level progressively decreases toward the distal end of the GI tract. Therefore, the distal GI tract is expected to be more severely affected in any condition that involves impaired contractility. Similarly, in the absence of urinary tract obstruction, the urinary bladder does not require maximal force for micturition and an apparent phenotype therefore may require a longer time period to develop. Myosin (*Myh11*)-driven Cre expression is considered to be highly smooth muscle–specific,^35^ and yet we noted expansion of the mucosal area in Y/T KO mice. This was not owing to reduced YAP and TAZ expression in any mucosal cell type because Cre-driven recombination events, as shown using reporter switching, were highly specific for smooth muscle cells. A more likely explanation is that distension of the colon, owing to the lack of force-generating ability, causes some level of mechanical damage to the mucosa and resultant activation of repair mechanisms. Transcripts for serum amyloid A1 (*Saa1*) and A2 (*Saa2*) were induced in Y/T KO mice, and this was not seen in the bladder which was not distended. *Saa1* and *Saa2* have been used in numerous prior studies as biomarkers of mucosal repair.^39^^,^^40^ In further support of mucosal repair, we noted additional mucosal markers among the induced transcripts in the colon such as *Lyz1* and *Alpi*.

The fraction of concordantly repressed transcripts in the colon and urinary bladder, as determined using RNA sequencing, was relatively small, being somewhere in the range of 10%–30%. This likely is owing in part to the stringent significance thresholds applied, causing some transcripts to just miss the threshold in one of the tissues, as illustrated by the M_3_ receptor, but it also may relate to the ability of YAP and TAZ to contribute to different transcriptional complexes depending on the tissue and cell type. The canonical DNA-binding partners of YAP and TAZ are TEA-domain transcription factors (TEAD1–4), but compelling studies also have shown that YAP and TAZ may interact with MRTFs.^23^^,^^25^^,^^41^ The latter are co-activators for SRF, which translocate to the nucleus in response to mechanical forces and actin dynamics, similar to YAP and TAZ. Whether the mutual dependence of MRTF-SRF and YAP-TAZ-TEAD signaling pathways is direct, as a result of physical binding, or indirect, mediated by cytoskeletal dynamics, remains an open question. However, it is clear from the present work and that of others^19^^,^^20^ that inducible smooth muscle–specific SRF and Y/T knockouts phenocopy each other, at least in regard to their GI phenotypes. Moreover, it is evident that concordantly repressed transcripts in the Y/T colon and bladder are enriched for classic MRTF-SRF targets, including *Acta2*, *Myh11*, and *Srf* itself.

One of the most definitive conceptual advances of this study is that the net impact of YAP and TAZ in vivo is to promote contractile function in colonic smooth muscle. The reason that this is important is that prior studies have suggested that activation of YAP (and TAZ) leads to loss of contractile smooth muscle markers.^42^ For instance, 1 study on arterial smooth muscle cells showed that YAP was up-regulated by platelet-derived growth factor in association with impaired binding of SRF to DNA, and reduced expression of contractile markers.^31^ Another study supported the view that YAP promotes smooth muscle growth and represses expression of contractile markers.^30^ It is possible that YAP and TAZ play different roles in GI and arterial smooth muscle, but it is perhaps more likely that some in vitro findings, relying on overexpression and silencing, respectively, fail to translate to the in vivo situation. Interestingly, deletion of YAP in vivo in smooth and cardiac muscle during embryonic development results in perinatal lethality associated with vascular abnormalities and distended arteries with reduced wall thickness.^26^ This suggests that mechanical properties of arteries are compromised by YAP/TAZ deletion, resulting in an inability to resist increased pressure levels.

Substrate stiffness activates YAP and TAZ,^6^ and, in certain models, such as pulmonary hypertension, this may elicit a vicious cycle in which YAP and TAZ, via various downstream mechanisms, further promote matrix stiffness.^43^, ^44^, ^45^ Our present results are in keeping with this overall paradigm because we find passive mechanical properties of the colon to be robustly altered. Passive force was reduced by well more than 80% at the largest circumferences, and much more than the thinning of the muscle layer (reduced by 40%). Such a drastic reduction of stiffness in only 12 days is staggering, especially considering the slow turnover of some matrix molecules. The reduced passive stiffness in Y/T KO colon likely is owing to a combination of intracellular changes in cytoskeletal organization and extracellular changes in matrix proteins. Gene ontology enrichment analysis of RNA sequencing data from Y/T KO colon support altered regulation of genes involved in matrix remodeling as well as a reduction in genes encoding cytoskeletal proteins. The increased diameter of the Y/T KO colon persisted after removal of the fecal content, suggesting an outward remodeling of the tissue. The smooth muscle layer was thinner in Y/T KO colon, but the diameter was increased, and hence the total smooth muscle area and cell number were unchanged. The fraction of proliferating smooth muscle cells was low (∼1%) in both control and Y/T KO, suggesting that the outward remodeling involves reorganization of existing smooth muscle cells around a larger lumen. The unchanged cell number and smooth muscle area argues against any effect on apoptosis at the early time points studied here. Furthermore, SRF KO mice only show increased apoptosis at 21 days after tamoxifen injections.^21^ Although it is likely that Y/T KO mice would show altered levels of proliferation, apoptosis, or smooth muscle cross-sectional area at later time points, we did not analyze mice past day 11 after tamoxifen treatment because of ethical constraints. At later time points, the mice suffer from malnutrition, which could affect the results independently of Y/T KO.

Taken together, the present report has shown that YAP and TAZ are guardians of colonic motility, and that their deletion in adult smooth muscle causes rapid lethality owing to colonic pseudo-obstruction. The impaired colonic motility is owing to compound effects on the expression of muscarinic receptors and contractile proteins. In addition, YAP and TAZ play essential roles in the maintenance of passive tissue stiffness in the distal GI tract. YAP and TAZ currently are considered appealing drug targets in cancer,^46^^,^^47^ but our findings underscore a need for cell-specific targeting strategies to avoid serious GI complications.

## Materials and Methods

All authors had access to the study data and reviewed and approved the final manuscript.

### Ethical Considerations

All experiments were approved by the Malmö/Lund animal ethics committee (M6-15 and M61-16) and adhere to the Animal Research Reporting of In Vivo Experiments guidelines.

### Animals

The TAZ/YAP floxed mice (*TAZ*^*fl/fl*^/*YAP*^*fl/fl*^) were purchased from Jackson Laboratory (Bar Harbor, ME) (*Wwtr1*^*tm1Hmc*^*/Yap1*^*tm1Hmc*^*/WranJ*; stock no: 030532, mixed background)^48^ and bred with hemizygous *Myh11-Cre/ERT2*^*Tg/0*^ male mice at Lund University, Sweden. The *Myh11-Cre/ERT2* mouse originally was provided by Stefan Offermanns (Max Planck Institute, Bad Nauheim, Germany), and has been described previously.^35^ Because the *Cre* transgene is located on the Y-chromosome, only male mice are positive for Cre. *TAZ*^*fl/fl*^/*YAP*^*fl/fl*^ females were bred with Cre-positive *TAZ*^*fl/fl*^/*YAP*^*fl/+*^ males. Cre-mediated recombination of floxed alleles was induced by intraperitoneal injection of tamoxifen (1 mg/mouse/day) dissolved in ethanol/sunflower seed oil (1:10) in 5- to 8-week-old male mice for 5 consecutive days. Tamoxifen-treated Cre-negative *TAZ*^*fl/fl*^/*YAP*^*fl/fl*^ male mice were used as controls unless otherwise stated. Animal code numbers were used to minimize subjective bias. Unless indicated, experiments were performed at 9 or 11 days after the first tamoxifen injection. mT/mG mice (B6.129[Cg]-*Gt*[*ROSA*]*26Sor*^*tm4*[*ACTB-tdTomato,-EGFP*]*Luo*^/J^29^) were used as Cre reporter strain and bred onto *TAZ*^*fl/+*^/*YAP*^*fl/fl*^*Myh11-Cre/ERT2* male mice. Tamoxifen-treated Cre-negative mT/mG *TAZ*^*fl/fl*^/*YAP*^*fl/fl*^ male mice were used as controls.

### Liquid Diet

For food consumption measurements, mice were introduced to a liquid diet (AIN-76, F1268; Bio-Serv, Frenchtown, NJ) in the presence of the standard solid diet for 3 days and then fed exclusively liquid diet for 12 days. Average food intake was measured daily for 4 mice per cage.

### Histologic Analysis

Segments of mouse colon were fixed overnight in 4% formaldehyde, rinsed in phosphate-buffered saline, dehydrated in a series of increasing ethanol concentrations, 2 changes of xylene, incubated in paraffin overnight, and then embedded in paraffin. Paraffin sections (5 μm) of mouse colon were stained using H&E (HistoLab, Västra Frölunda, Gothenburg). Slides were mounted using Pertex mounting medium (HistoLab) and visualized with the Leica/Aperio ScanScope systems (Buffalo Grove, IL). The cross-sectional area was measured using Adobe Photoshop CC 2018 (San Jose, CA).

### Immunohistochemical Analysis

For immunohistochemical analysis, antigen retrieval was performed in citrate buffer, pH 6.0 (C9999, 1000 mL; Sigma-Aldrich, St. Louis, MO) for 20 minutes. After washing in TBS-T, sections were permeabilized with 0.1% Triton X-100 for 10 minutes at room temperature. Blocking was performed in 3% bovine serum albumin (Sigma-Aldrich) + 0.3 mol/L glycine (50046; Sigma-Aldrich) for 1 hour at room temperature, followed by incubation with the primary antibody against YAP (4912, 1:400; Cell Signaling Technology, Danvers, MA) and TAZ (ab84927, 1:250; abcam, Cambridge, England) at 4°C overnight. Primary antibodies were diluted in 3% bovine serum albumin. Endogenous peroxidases were blocked with 4% H_2_O_2_ in methanol for 20 minutes at room temperature. After washing, sections with YAP antibody were incubated with mouse- and rabbit-specific horseradish peroxidase (HRP)/3,3′-diaminobenzidine tetra hydrochloride detection immunohistochemistry kit (abcam, ab64263) according to the manufacturer’s instructions. Sections with TAZ antibody were incubated with anti-rabbit HRP-conjugated secondary antibody (Cell Signaling Technology, 7074P2) for 30 minutes at room temperature in 3% bovine serum albumin. After washing, sections were incubated with 3,3′-diaminobenzidine tetra hydrochloride for 2 minutes. Specific primary antibodies were omitted in negative controls of the reactions. Nuclei were counterstained with Mayer’s hematoxylin (01820; HistoLab). Slides were mounted using xylene mounting medium (00840; HistoLab) and images were captured using BX60 microscope (Olympus, Tokyo, Japan).

### Fluorescence Microscopy

Cryosections (8 μm) of mouse colon were labeled with Ki67 (rabbit polyclonal, 1:200, abcam), counterstained with DAPI, and visualized with an Olympus BX60 microscope using cellSens Dimension software (Olympus). ImageJ was used to count Ki67-positive cells and the total number of nuclei (DAPI) in the smooth muscle layer. Primary antibodies were detected with relevant secondary antibodies (Invitrogen, Carlsbad, CA). For detection of F-actin and G-actin, cryosections were incubated with Alexa Fluor–633 phalloidin and DNaseI (1:200; Invitrogen), respectively, and counterstained with DAPI. Slides were mounted using Aqua PolyMount medium (Polysciences, Inc, Warrington, PA) and images were captured using a Nikon A1+ confocal system with NIS-Elements software (Nikon instruments, Melville, NY).

### RNA Sequencing

Total RNA from colon and bladder homogenates was isolated using the RNeasy Mini kit (74104; Qiagen, Germantown, MD) in a QIAcube (Qiagen) according to the manufacturer’s instructions. RNA concentration and purity were assessed using a NanoDrop spectrophotometer (ND 2000; Thermo Scientific, Waltham, MA). Samples were checked for RNA quality using a 2100 Bioanalyzer instrument (Agilent, Santa Clara, CA). Samples with an RNA integrity number value greater than 7 (RNA integrity number values, means ± SEM: colon, 8.4 ± 0.15; bladder, 7.98 ± 0.21) were used for library preparations (TruSeq; Illumina, San Diego, CA) and subjected to RNA-seq analysis using the NextSeq 500 platform (Illumina). Expression levels of each gene and transcript were analyzed using StringTie and differentially expressed gene analysis by DESeq2. The data have been deposited in NCBI's Gene Expression Omnibus^37^ and are accessible through GEO Series, accession number: GSE 150603 (https://www.ncbi.nlm.nih.gov/geo/query/acc.cgi?acc=GSE150603).

Gene ontology enrichment analysis was performed by the protein analysis through evolutionary relationships Overrepresentation Test (released April 7, 2020) using the Gene ontology database (released June 1, 2020).^49^, ^50^, ^51^ The Fisher exact test and correction for false discovery rate were used for statistical analysis.

### Real-Time, Reverse-Transcription Polymerase Chain Reaction

Whole colon pieces were excised and cleaned in HEPES-buffered Krebs solution (Ca^2+^-free). They were either snap frozen in liquid nitrogen or dissected under the microscope to remove mucosa by fine forceps. The samples were disrupted and homogenized by TissueLyser LT (85600; Qiagen) in the presence of stainless steel beads, then QIAzol was added. Isolation of total RNA and quantitative polymerase chain reaction was performed as described previously.^52^ Commercially available primers from QuantiTect Primer assays (Qiagen) were used.

### Protein Extraction and Western Blot

Preparation and Western blot of colon samples homogenates was performed as described previously.^53^ After transfer, total protein content was assayed using the Revert 700 Total Protein Stain kit (926-11010; LI-COR Biosciences, Lincoln, NE). Specific proteins were detected using commercially available primary antibodies: YAP/TAZ (cat. 8418, 1:1000; Cell Signaling Technology), α-actin (cat. A5228, 1:1000; Sigma-Aldrich), smooth muscle myosin heavy chain (cat. ab53219, 1:1000; abcam), Chrm2 (cat. AMR-002, 1:1000; alomone labs, Jerusalem), Chrm3 (cat. AMR-006, 1:200; alomone labs), SRF (cat. 5147S, 1:1000; Cell Signaling Technology), and myosin light chain (3672S, 1:1000; Cell Signaling Technology). HRP-conjugated or fluorescently labeled secondary antibodies were used and images were acquired using the Odyssey Fc instrument and analyzed using Image Studio software (LI-COR Biosciences).

### Wire Myography

Two rings of the descending colon from each mouse were dissected from the surrounding tissues and cleaned. Colon rings were mounted in wire myographs (Danish Myo Technology, Aarhus, Denmark). The preparations were kept in HEPES-buffered Krebs solution (135.5 mmol/L NaCl, 5.9 mmol/L KCl, 1.2 mmol/L MgCl_2_, 11.6 mmol/L HEPES, 11.5 mmol/L glucose, and 143.8 mmol/L Cl^-^), pH 7.35 at 37°C. Preparations were allowed to equilibrate at 2 mN basal tension for 30 minutes in 2.5 mmol/L Ca^2+^-HEPES–buffered Krebs solution before the first stimulation with 60 mmol/L KCl. CCh (CAS51-83-2; Sigma-Aldrich) was added in a cumulative manner and the integrated force for 2 minutes was calculated for each concentration to obtain the dose-response curve. For the length–tension relationship, colon rings were set to 5.9 mm where the passive force is zero, then stretched to a specific internal circumference and allowed to equilibrate in Ca^2+^-containing buffer before contraction with 60 mmol/L KCl. After 7 minutes of stimulation, the solutions were changed to Ca^2+^ free buffer and the passive force was measured by integrating force for 10 minutes. The active force was calculated by subtracting the passive force from the integrated (total) force for 7 minutes with KCl (see Figure 6*E* for further details). All force measurements were normalized to the width of corresponding preparation, which was determined under the dissection microscope.

### Correlation Analysis Using the GTExPortal Repository

RNA sequencing data were downloaded from the GTExPortal.org in June 2020 using R-scripts as described.^52^ Correlations of transcript levels (in transcripts per million) were examined using the Spearman method in GraphPad Prism (San Diego, California). Five gastrointestinal organs and the urinary bladder were examined. The number of individuals per tissue ranged from 21 (bladder) to 406 (colon transverse). *P* < .0001 was considered significant. The Ensembl identifiers were as follows: ENSG00000137693.13_YAP1, ENSG00000141052.17_MYOCD, ENSG00000196588.14_MKL1, ENSG00000112658.7_SRF, ENSG00000181072.11_CHRM2, ENSG00000133019.11_CHRM3, ENSG00000007866.19_TEAD3, and ENSG00000018408.14_WWTR1.

### Statistical Analysis

Results are presented as means ± SEM. Statistical significance was determined using the Student *t* test, the Mann–Whitney test, or 2-way analyses of variance, followed by the Bonferroni post hoc test for multiple comparisons using GraphPad Prism Software version 5 and 8.
