# Supplementary material for: Inducible Deletion of YAP and TAZ in Adult Mouse Smooth Muscle Causes Rapid and Lethal Colonic Pseudo-Obstruction
Source: Cell Mol Gastroenterol Hepatol. 2020 Sep 28;11(2):623–37. doi: 10.1016/j.jcmgh.2020.09.014 (PMC7806867; doi:10.1016/j.jcmgh.2020.09.014)
Supplement: Supplementary Table [file mmc1.pdf]

Supplementary Table 1. Significantly upregulated and downregulated mRNA transcripts in colon or bladder from YAP/TAZ KO vs control mice. Expression of mRNA transcripts was analyzed by RNA-sequencing one week post induction. Adjusted p-values was determined using DESeq2 analysis. Red text indicates that the transcripts are differentially regulated in both colon and bladder.

Downregulated genes in bladder		
padj	Gene	(FC) KO
1,42E-02	*Ifi202b*	0,006
1,51E-15	*Npy6r*	0,007
7,21E-03	*Ggcx*	0,012
1,15E-05	*Slc6a16*	0,022
8,20E-08	*Sec1*	0,031
3,57E-02	*Gm9008*	0,034
3,04E-03	*A2m*	0,056
4,90E-02	*Tmem150a*	0,064
2,62E-04	*Klk6*	0,071
3,31E-07	*Itgb3bp*	0,074
1,77E-02	*Acod1*	0,082
**2,77E-02**	***Bdnf***	**0,091**
4,88E-02	*Traf5*	0,098
**5,60E-07**	***Kcnf1***	**0,098**
**7,39E-15**	***Ptprz1***	**0,115**
8,84E-05	*Slc1a1*	0,119
2,71E-02	*Gm5483*	0,127
3,04E-03	*Slc17a7*	0,133
5,53E-04	*Grin2c*	0,136
2,49E-03	*Tnn*	0,151
**8,80E-04**	***Tent5b***	**0,159**
3,85E-04	*Zfp473*	0,160
3,81E-02	*Gm3512*	0,160
4,80E-02	*Cxcl3*	0,169
**4,03E-06**	***Kif26a***	**0,172**
1,56E-02	*Stfa2l1*	0,184
6,67E-03	*Slfn4*	0,193
**1,33E-02**	***Slc26a7***	**0,194**
9,66E-04	*S100g*	0,219
**3,95E-12**	***Igfbp2***	**0,221**
**2,47E-13**	***Fhl1***	**0,223**
1,37E-06	*Htr1b*	0,228
9,62E-04	*Fpr1*	0,231
2,42E-05	*Krt14*	0,235
8,00E-05	*Chrdl2*	0,242
**1,07E-12**	***Tnnt2***	**0,249**
**1,44E-04**	***Hcrtr1***	**0,251**
2,87E-02	*Il1b*	0,259
**2,26E-14**	***Mmp17***	**0,262**
**1,03E-03**	***Chrm2***	**0,265**
4,11E-06	*Junb*	0,269
1,86E-02	*Rnf212*	0,274
3,31E-07	*Grin2d*	0,293
8,95E-05	*Nell1*	0,296
4,90E-02	*Pthlh*	0,300

Downregulated genes in colon		
padj	Gene	(FC) KO
7,44E-04	*Reg1*	0,001
2,57E-05	*Amy2a3*	0,002
4,96E-03	*Reg2*	0,002
1,37E-03	*Try4*	0,003
7,56E-04	*Cela3b*	0,003
1,35E-02	*Try5*	0,003
4,18E-02	*Reg3a*	0,005
7,68E-10	*Cela2a*	0,006
1,65E-04	*Prss2*	0,007
1,60E-04	*2210010C04Rik*	0,007
1,49E-05	*Cel*	0,009
1,30E-03	*Cpa1*	0,010
2,59E-02	*Pnlip*	0,012
1,13E-02	*Amy2a4*	0,012
1,19E-02	*Cuzd1*	0,012
1,65E-02	*Ctrc*	0,014
5,11E-04	*Crisp1*	0,018
3,84E-03	*Ctrb1*	0,038
3,32E-16	*Ankrd1*	0,056
1,08E-02	*Chst5*	0,057
**7,37E-27**	***Igfbp2***	**0,067**
5,97E-03	*Rnf181*	0,079
**8,32E-03**	***Slc26a7***	**0,087**
1,59E-04	*Alkal1*	0,094
**7,84E-06**	***Hcrtr1***	**0,101**
9,25E-04	*Col6a6*	0,136
5,46E-28	*Ctxn3*	0,144
**1,00E-16**	***Tnnt2***	**0,156**
3,49E-05	*Gypa*	0,158
7,40E-05	*Colec10*	0,167
7,00E-04	*Hsd3b1*	0,169
**4,60E-05**	***Ptprz1***	**0,189**
**5,24E-03**	***Cdkl1***	**0,191**
**7,04E-14**	***Tent5b***	**0,192**
**1,23E-04**	***Bdnf***	**0,194**
**5,55E-03**	***Kcnn2***	**0,196**
2,97E-03	*Cpb1*	0,208
**3,48E-03**	***Ooep***	**0,209**
5,68E-09	*Angptl7*	0,218
**2,95E-09**	***Chrm2***	**0,219**
5,11E-05	*Rgs1*	0,229
1,85E-06	*2310079G19Rik*	0,236
4,86E-02	*Hs3st5*	0,237
8,11E-15	*Mal*	0,248
3,48E-03	*Ly6g6c*	0,267

1,68E-02	*Nrgn*	0,303
1,12E-02	*Prkg2*	0,305
1,62E-11	*Car11*	0,308
4,43E-02	*Brca1*	0,309
7,47E-03	*Nptx1*	0,310
4,08E-02	*Atp6v0a4*	0,332
3,23E-05	*Neto2*	0,333
4,98E-04	*Gm10282*	0,336
6,49E-04	*Tnfrsf11b*	0,338
4,22E-02	*Wisp2*	0,350
5,60E-07	*Col8a2*	0,350
7,84E-03	*CAAA01147332.1*	0,353
4,46E-04	*Myl4*	0,360
5,43E-03	*Cit*	0,368
1,26E-07	*Actn1*	0,369
2,92E-06	*Tinagl1*	0,372
3,78E-02	*Stra6*	0,372
7,52E-16	*Serpinh1*	0,372
4,88E-02	*Insc*	0,372
1,21E-04	*Kcng4*	0,374
7,52E-04	*Fblim1*	0,377
3,37E-02	*Car9*	0,377
1,44E-02	*6820408C15Rik*	0,379
9,21E-03	*Slc39a14*	0,382
2,61E-02	*Ccl7*	0,382
3,21E-02	*Cck*	0,388
4,92E-02	*Lrp2*	0,388
**3,16E-05**	***Lims2***	**0,391**
**1,65E-02**	***Ooep***	**0,397**
4,10E-03	*Foxs1*	0,398
**1,29E-03**	***Tgm3***	**0,399**
5,47E-06	*Fstl3*	0,402
3,03E-03	*Kifc3*	0,403
1,71E-03	*Uhrf1*	0,404
3,30E-02	*Dtl*	0,404
1,33E-02	*Plaur*	0,405
8,82E-07	*Chrm3*	0,406
5,24E-03	*1700061G19Rik*	0,407
1,21E-03	*Il22ra1*	0,407
**3,82E-05**	***Flnc***	**0,409**
3,09E-03	*Zbtb8b*	0,409
4,73E-02	*Myo7a*	0,417
1,52E-02	*Plekhg2*	0,417
2,61E-02	*Socs3*	0,418
1,09E-02	*C1rb*	0,419
1,79E-02	*B3gnt5*	0,424
7,34E-03	*Rasl11a*	0,425
1,61E-03	*Ncf4*	0,426

7,18E-03	*Higd1c*	0,307
4,68E-03	*Adipoq*	0,308
3,38E-02	*Dpp10*	0,311
1,00E-09	*Trpm6*	0,317
4,67E-03	*Riiad1*	0,319
**2,75E-06**	***Gm11627***	**0,321**
1,35E-02	*Bmp8b*	0,331
**7,24E-07**	***Lims2***	**0,334**
1,19E-02	*Retn*	0,340
5,17E-10	*Akr1b7*	0,346
7,35E-10	*Nov*	0,363
**7,74E-03**	***Kcnf1***	**0,374**
6,96E-10	*Ptn*	0,375
5,24E-03	*Tph1*	0,375
1,25E-03	*Gdnf*	0,379
3,23E-02	*Methig1*	0,385
1,67E-02	*Agr3*	0,388
4,50E-04	*Cpm*	0,390
3,76E-02	*Rhov*	0,396
**1,89E-03**	***Wscd2***	**0,399**
5,36E-04	*Asb11*	0,400
**1,22E-02**	***Adam33***	**0,403**
1,44E-02	*Zfp791*	0,405
7,46E-03	*Klb*	0,407
7,84E-06	*Pla2g3*	0,407
**1,62E-06**	***Hhip***	**0,407**
1,74E-02	*P2ry4*	0,420
4,75E-03	*Dtna*	0,422
7,97E-03	*Lrch2*	0,424
3,23E-02	*Hist1h1e*	0,425
1,84E-03	*Nipal4*	0,431
7,60E-03	*Car1*	0,432
1,94E-02	*Ppil6*	0,433
**2,28E-02**	***Kif26a***	**0,443**
5,04E-03	*Slc30a10*	0,447
1,65E-02	*Btbd8*	0,454
**7,60E-03**	***Atp2b4***	**0,465**
7,29E-06	*Pmaip1*	0,468
1,68E-02	*Rnf144b*	0,475
1,17E-03	*Rab3b*	0,477
4,94E-03	*2210407C18Rik*	0,480
1,73E-05	*Synpo2*	0,481
**1,56E-05**	***Msl3***	**0,488**
4,94E-03	*Ces2c*	0,497
**3,48E-03**	***Flnc***	**0,503**
4,07E-02	*Popdc2*	0,505
**2,97E-02**	***Id4***	**0,510**
1,89E-03	*Aldh1a1*	0,510

**2,14E-14**	***Id4***	**0,429**
5,08E-03	*Klhdc8a*	0,429
**6,56E-04**	***Actg2***	**0,429**
3,44E-02	*Lyrm7*	0,430
1,10E-04	*Ppp1r3b*	0,431
2,38E-03	*Asf1b*	0,431
1,00E-02	*Nacad*	0,433
4,20E-06	*Cgref1*	0,433
8,02E-09	*Des*	0,433
3,87E-02	*Tnfsf9*	0,437
7,50E-03	*Krt6a*	0,437
3,38E-03	*Itgb8*	0,441
3,86E-05	*Mcm5*	0,441
7,45E-06	*Sorbs1*	0,444
1,09E-02	*Cdt1*	0,447
4,15E-02	*Cep72*	0,448
3,57E-02	*Gm27021*	0,448
8,65E-05	*Pdlim7*	0,450
**1,60E-13**	***Cav1***	**0,451**
3,55E-02	*Ppfia3*	0,452
2,43E-02	*Aldh1l2*	0,452
5,95E-03	*Fcgr4*	0,454
1,25E-02	*Pim1*	0,456
2,81E-02	*Gjb5*	0,456
7,04E-12	*Yap1*	0,456
1,77E-05	*Dok4*	0,457
4,07E-03	*Lrrc10b*	0,459
1,06E-05	*Gpr4*	0,461
3,73E-05	*Itga5*	0,464
1,06E-07	*Cald1*	0,464
2,48E-02	*Cetn4*	0,467
2,87E-03	*Ccdc88b*	0,471
7,71E-05	*Cav3*	0,472
4,34E-03	*Ephx3*	0,472
6,83E-03	*Midn*	0,472
**3,87E-05**	***Wscd2***	**0,474**
1,16E-02	*Ccl12*	0,476
**1,03E-03**	***Gm11627***	**0,476**
4,59E-07	*Spon2*	0,476
2,54E-03	*Ror2*	0,481
3,82E-02	*Gpm6a*	0,482
2,30E-04	*Eno1*	0,491
**1,71E-21**	***Msl3***	**0,500**
3,42E-06	*Nosip*	0,500
1,96E-02	*Ak4*	0,501
3,87E-02	*Dok2*	0,502
2,12E-05	*Lmnb2*	0,503
2,10E-02	*Loxl4*	0,503
**3,95E-03**	***Tgm3***	**0,510**
7,96E-03	*Ace2*	0,514
1,49E-02	*Scd1*	0,515
1,53E-05	*Myh11*	0,517
1,13E-02	*Ogn*	0,521
**8,20E-03**	***Mmp17***	**0,521**
1,79E-02	*Slc26a3*	0,522
1,74E-02	*Qpct*	0,523
1,89E-03	*Nt5c3*	0,523
1,13E-02	*Cyp2c55*	0,537
1,28E-02	*Kbtbd11*	0,537
**4,99E-05**	***Acta2***	**0,538**
**6,67E-03**	***Plekhg6***	**0,540**
2,89E-02	*Adamts8*	0,544
1,54E-02	*Abcb1a*	0,545
**2,47E-02**	***Notch3***	**0,548**
1,78E-02	*H2-Q10*	0,552
3,43E-02	*Adamtsl1*	0,553
**7,94E-03**	***Cav1***	**0,553**
3,11E-03	*Arntl*	0,555
**3,12E-04**	***Fhl1***	**0,557**
1,82E-02	*Zfp775*	0,557
2,74E-02	*Mep1a*	0,561
1,71E-02	*Plin4*	0,577
2,18E-02	*Vgll3*	0,579
9,54E-03	*Fbxl22*	0,582
**3,34E-02**	***H2-Q1***	**0,584**
**3,25E-04**	***Actg2***	**0,587**
**1,17E-02**	***Synm***	**0,587**
1,06E-02	*Cryab*	0,588
1,13E-02	*Phlpp2*	0,589
3,38E-02	*Slc35e3*	0,593
2,04E-02	*Aqp8*	0,594
4,50E-02	*Aspn*	0,600
3,76E-02	*Ugdh*	0,600
4,31E-02	*Cited2*	0,601
**3,78E-02**	***Fzd2***	**0,601**
4,32E-02	*Entpd5*	0,605
1,71E-02	*Ces2b*	0,606
**1,22E-02**	***Cdc42ep3***	**0,618**
1,54E-02	*Rgs5*	0,621
1,32E-02	*Car2*	0,629
4,68E-03	*Atp2b1*	0,630
4,86E-02	*Rgs4*	0,631
1,19E-02	*Myl9*	0,634
4,09E-02	*Casp3*	0,638
2,07E-02	*Bmp5*	0,648
3,81E-02	*Vps13c*	0,649

4,08E-02	*Padi4*	0,504
1,66E-03	*Ada*	0,504
**1,42E-02**	***Kcnn2***	**0,505**
5,91E-03	*Aldh16a1*	0,507
3,61E-03	*Pitx1*	0,507
1,88E-03	*Adamts9*	0,508
6,78E-03	*Ildr2*	0,508
2,42E-02	*Bcl3*	0,511
4,61E-02	*Ltc4s*	0,511
1,20E-06	*Wfdc1*	0,512
1,29E-03	*Gm49450*	0,512
7,09E-04	*Col12a1*	0,512
**4,76E-05**	***Cdc42ep3***	**0,516**
3,76E-03	*Pcolce*	0,516
4,74E-03	*Slc16a3*	0,516
4,38E-02	*Slc15a3*	0,517
8,90E-04	*Hic1*	0,517
1,82E-02	*Wnt11*	0,519
1,26E-07	*Pkdcc*	0,520
4,92E-03	*Gins2*	0,523
7,86E-06	*Col25a1*	0,525
7,47E-03	*Tppp3*	0,526
8,65E-05	*Nfkb2*	0,528
**3,87E-02**	***Adam33***	**0,529**
2,84E-02	*Map3k14*	0,533
2,67E-03	*Clec11a*	0,533
3,51E-03	*Prr11*	0,533
1,77E-05	*Hjurp*	0,536
2,75E-02	*Rhbdf1*	0,536
4,36E-02	*Il18r1*	0,537
8,64E-03	*Tead2*	0,538
5,83E-06	*Chpf*	0,539
2,75E-05	*Tmem132a*	0,539
2,27E-04	*Tubb6*	0,542
1,09E-02	*Mif*	0,544
1,29E-02	*Adam19*	0,546
3,07E-04	*Ppp1r12c*	0,548
4,48E-02	*Ccl5*	0,548
3,50E-02	*Prr15*	0,549
1,01E-02	*Lurap1l*	0,549
2,43E-02	*Adamts7*	0,549
6,61E-03	*Gm5617*	0,551
1,02E-02	*Coro1a*	0,555
7,33E-03	*Crip1*	0,555
7,33E-03	*Itga3*	0,556
2,05E-03	*Wdr1*	0,557
9,21E-03	*Aldh1a2*	0,557
2,57E-02	*Ces2e*	0,559

2,25E-02	*Aoc3*	0,650
**4,83E-02**	***Tpm1***	**0,678**
2,14E-02	*Filip1l*	0,687
**3,49E-02**	***Tagln***	**0,696**

8,47E-03	*Ndufa4l2*	0,559
2,73E-03	*Hspb2*	0,559
1,37E-08	*Id3*	0,559
2,54E-03	*Tes*	0,560
3,94E-02	*N4bp3*	0,560
7,35E-03	*Pfkp*	0,561
3,14E-04	*Hspb1*	0,562
1,37E-07	*Krt5*	0,562
2,96E-05	*Tuba1b*	0,563
**6,61E-03**	***Acta2***	**0,567**
3,89E-03	*Klc2*	0,568
3,14E-04	*Rhbdf2*	0,568
2,61E-03	*Prss23*	0,568
2,37E-02	*Col27a1*	0,569
3,47E-02	*Fn1*	0,570
4,18E-02	*Ptgis*	0,571
4,26E-03	*Ptprs*	0,572
1,00E-04	*Crip2*	0,573
1,18E-02	*Napsa*	0,574
1,79E-02	*Pfkl*	0,577
2,59E-02	*Hs3st1*	0,578
**1,89E-03**	***Tagln***	**0,579**
3,62E-04	*Ccnd3*	0,580
1,19E-08	*Elob*	0,582
4,80E-02	*Kcne4*	0,583
7,55E-03	*Bdh1*	0,583
6,61E-03	*Gm13889*	0,584
8,19E-05	*Polr2l*	0,584
7,35E-03	*Dagla*	0,586
4,19E-02	*Afap1*	0,586
5,67E-04	*Slc16a1*	0,587
1,80E-03	*Piezo1*	0,587
4,29E-04	*Myocd*	0,589
4,20E-02	*Crlf2*	0,589
4,39E-02	*Mmp28*	0,589
9,50E-03	*Fads3*	0,589
1,58E-02	*Gbp2*	0,589
2,42E-02	*Col1a1*	0,590
3,42E-06	*Col5a3*	0,591
8,65E-05	*Zbtb7a*	0,591
6,43E-06	*Lmna*	0,592
1,33E-02	*Pole2*	0,594
4,95E-04	*Sorbs2*	0,594
5,73E-03	*Rexo1*	0,596
**8,00E-05**	***Atp2b4***	**0,596**
9,25E-05	*Dapk3*	0,596
9,38E-08	*Susd2*	0,596
2,19E-02	*Rdh10*	0,598

1,71E-02	*Slc9a3r1*	0,598
3,50E-02	*Trip6*	0,600
2,00E-03	*Zmiz1*	0,600
3,09E-02	*Plppr5*	0,600
2,91E-03	*Nfic*	0,601
3,82E-02	*Vtcn1*	0,601
4,90E-02	*Slc27a3*	0,601
8,36E-03	*Myo1c*	0,602
5,86E-03	*Nab2*	0,603
3,77E-02	*Syndig1*	0,603
2,59E-02	*Aifm2*	0,603
4,26E-03	*Pdgfrl*	0,604
2,56E-03	*P4ha1*	0,604
3,74E-02	*Trmt61a*	0,606
4,36E-05	*P3h1*	0,606
5,24E-03	*2200002D01Rik*	0,608
**3,82E-02**	***Fzd2***	**0,609**
2,55E-02	*Nkd2*	0,613
4,39E-02	*Ptrh1*	0,613
3,37E-02	*Tln1*	0,613
1,30E-02	*Slc2a1*	0,613
4,21E-02	*Prss22*	0,615
5,04E-05	*Zfp651*	0,615
4,79E-03	*Stk40*	0,616
2,73E-02	*2310022B05Rik*	0,616
3,79E-02	*Ccdc102a*	0,617
1,42E-02	*Ppp1r18*	0,617
5,35E-04	*Srf*	0,618
2,05E-05	*Col15a1*	0,618
3,80E-03	*Ccdc124*	0,620
7,35E-04	*Sdc3*	0,621
4,49E-02	*Zbtb7c*	0,622
2,22E-02	*Tmem107*	0,623
7,34E-03	*Nt5dc2*	0,623
3,38E-04	*Nrp2*	0,624
6,24E-03	*Creb3l1*	0,624
1,66E-02	*Tagln2*	0,624
2,82E-02	*Syde1*	0,625
4,22E-02	*Rgs19*	0,626
3,09E-02	*Osr1*	0,626
3,16E-02	*Sox18*	0,626
3,37E-03	*Proser2*	0,626
3,70E-02	*Rps12-ps3*	0,627
3,38E-02	*Vcan*	0,627
**4,59E-02**	***Cdkl1***	**0,628**
3,65E-03	*Fam129a*	0,630
1,97E-03	*Fkbp10*	0,630
1,50E-02	*Rpl36*	0,631

1,95E-02	*Prkcb*	0,631
4,02E-02	*Hmgb1*	0,631
3,20E-02	*Ppp1r13l*	0,632
9,97E-03	*Chaf1a*	0,633
1,68E-02	*Sostdc1*	0,633
7,33E-03	*Hspbp1*	0,633
**3,33E-09**	***Notch3***	**0,633**
9,53E-04	*Vcl*	0,633
1,83E-02	*Il4ra*	0,633
2,82E-02	*Sbno2*	0,636
1,43E-02	*Lgals1*	0,637
3,65E-03	*Col5a1*	0,638
2,90E-02	*Tgm2*	0,639
1,29E-03	*Rai1*	0,639
1,78E-04	*Tubb4b*	0,639
**1,85E-03**	***Synm***	**0,639**
3,45E-05	*Gm11127*	0,640
1,53E-02	*Atp10a*	0,641
3,04E-02	*Rock2*	0,642
5,19E-03	*Qsox1*	0,643
4,16E-02	*Slc12a4*	0,645
**2,91E-02**	***H2-Q1***	**0,646**
4,35E-02	*Tns4*	0,646
1,76E-02	*Nr2f6*	0,647
7,95E-03	*Anxa2*	0,647
3,44E-02	*CAAA01118383.1*	0,648
3,89E-02	*Snai1*	0,649
2,75E-02	*Col16a1*	0,650
1,00E-02	*Dpm3*	0,650
4,36E-02	*Nek6*	0,650
3,57E-03	*Pih1d1*	0,650
4,22E-02	*Soga1*	0,650
4,76E-05	*Ehd2*	0,651
1,36E-02	*Sorcs2*	0,651
1,60E-02	*Col6a2*	0,652
2,67E-03	*Rara*	0,653
3,38E-04	*Trim8*	0,653
4,40E-02	*Bok*	0,654
1,02E-02	*Srebf2*	0,655
2,92E-02	*Trim47*	0,655
2,48E-02	*Frmd4a*	0,655
1,56E-03	*Bcl9l*	0,657
2,36E-03	*Sipa1*	0,657
1,97E-02	*Aldh3b1*	0,658
2,48E-03	*Hmga1*	0,659
2,98E-02	*Arhgef1*	0,659
4,62E-02	*Plec*	0,660
5,83E-03	*Myl6*	0,660

4,97E-09	*Rplp1*	0,660
4,07E-03	*Cactin*	0,662
1,95E-02	*Itga1*	0,662
4,08E-02	*Cnn1*	0,662
6,61E-03	*Efemp2*	0,662
1,12E-02	*Kcnn3*	0,663
8,12E-03	*Steap2*	0,663
1,08E-02	*Gas2l1*	0,663
2,50E-02	*1810055G02Rik*	0,663
9,81E-03	*Tubb5*	0,665
4,42E-02	*Cd44*	0,666
1,07E-04	*Efnb1*	0,666
3,05E-05	*Gpr153*	0,667
9,39E-03	*Col4a1*	0,668
1,42E-02	*Bves*	0,668
8,90E-04	*Yif1b*	0,668
4,01E-02	*Actb*	0,669
2,98E-02	*Icam1*	0,669
1,68E-02	*Hivep3*	0,671
2,56E-02	*Plekhh3*	0,671
4,82E-04	*Dynll1*	0,672
1,15E-03	*Sf3b5*	0,672
3,55E-02	*Tnrc18*	0,672
1,11E-04	*Tesk1*	0,673
1,15E-04	*Pawr*	0,675
4,21E-02	*Fkbp14*	0,675
3,54E-02	*Lmnb1*	0,675
2,84E-02	*Bak1*	0,676
1,79E-02	*Atn1*	0,676
1,22E-02	*Mrgprf*	0,677
4,83E-02	*Myh14*	0,678
4,22E-02	*Arhgef10l*	0,678
6,92E-03	*Polr2f*	0,680
4,12E-02	*Shmt2*	0,680
2,01E-02	*H2afy2*	0,681
1,30E-02	*Pgm5*	0,682
3,09E-02	*Gpaa1*	0,682
3,63E-02	*Pigyl*	0,682
1,68E-02	*Map7d1*	0,682
1,18E-02	*Bcar1*	0,683
1,01E-02	*Tcf3*	0,683
1,35E-03	*Mid1ip1*	0,684
3,97E-03	*Bysl*	0,686
1,19E-02	*Vps37b*	0,687
6,89E-04	*Pofut2*	0,688
1,74E-02	*Jak3*	0,688
4,02E-02	*Tnk2*	0,688
4,83E-02	*Dot1l*	0,690

4,85E-02	*Tmem143*	0,690
2,82E-02	*Tpp1*	0,690
7,52E-03	*H2-K1*	0,691
1,16E-02	*Dpp9*	0,691
5,40E-03	*Hspa12b*	0,694
2,61E-02	*Aldh18a1*	0,694
8,84E-03	*Snrpa*	0,695
**1,13E-02**	***Tpm1***	**0,695**
4,02E-02	*Itgb4*	0,695
9,21E-03	*Spsb2*	0,697
2,41E-03	*Itih5*	0,697
3,68E-02	*Mcm4*	0,697
4,96E-02	*Ncln*	0,699
4,35E-02	*Ubtd1*	0,700
3,39E-02	*Bola2*	0,700
7,09E-04	*Ptbp1*	0,701
3,09E-02	*Znrf3*	0,701
4,49E-02	*Ahcy*	0,702
4,17E-03	*Loxl1*	0,702
1,12E-02	*Krt7*	0,703
3,91E-02	*Mvk*	0,704
2,79E-02	*Cspg4*	0,704
2,82E-02	*Spire2*	0,705
4,88E-02	*Pxn*	0,705
2,58E-02	*P3h4*	0,706
2,69E-02	*Tuba1c*	0,706
**1,50E-04**	***Hhip***	**0,707**
4,52E-02	*Glipr2*	0,707
8,08E-03	*Dazap1*	0,707
4,88E-02	*Wnt4*	0,707
3,32E-02	*Lzts2*	0,707
2,71E-02	*Dohh*	0,707
3,37E-02	*Vegfa*	0,708
3,73E-02	*Reep4*	0,708
4,62E-02	*Syt8*	0,708
4,19E-02	*Coro1c*	0,709
1,64E-05	*Uqcc2*	0,710
2,78E-02	*Micall1*	0,711
1,22E-02	*Gpr137*	0,711
2,91E-02	*Pmepa1*	0,711
2,53E-02	*Triobp*	0,711
1,73E-02	*Atf5*	0,712
2,31E-02	*Vps18*	0,713
6,43E-06	*Ckap4*	0,713
1,61E-03	*Gys1*	0,713
9,32E-03	*Tkfc*	0,715
2,28E-02	*Pcnx3*	0,715
3,69E-02	*Fgl2*	0,715

2,53E-02	*Psmb10*	0,715
8,12E-03	*Gipc1*	0,716
3,44E-02	*Actg1*	0,716
2,96E-02	*Dusp18*	0,717
1,19E-02	*S100a10*	0,717
3,57E-02	*Gm48551*	0,718
1,33E-02	*Timm13*	0,719
2,28E-02	*Pgam1*	0,719
4,27E-02	*Gfra2*	0,719
1,74E-02	*Cd248*	0,721
7,33E-03	*Raver1*	0,721
1,53E-04	*Rplp2*	0,721
1,19E-02	*Emd*	0,722
5,84E-03	*Ndufb7*	0,722
1,12E-02	*Lgals3bp*	0,722
2,65E-02	*Evpl*	0,723
9,21E-03	*Snrpb*	0,725
1,61E-02	*Alad*	0,725
2,56E-03	*Ptprf*	0,726
2,82E-02	*Ywhag*	0,727
9,98E-03	*Ivl*	0,727
1,46E-02	*Ctbp1*	0,728
7,98E-03	*Romo1*	0,728
4,08E-02	*Cln8*	0,728
7,50E-03	*Arpc1b*	0,729
2,80E-02	*Tnks1bp1*	0,729
3,12E-02	*Galk1*	0,729
3,45E-03	*Uqcr11*	0,732
1,12E-02	*Atp5d*	0,732
2,82E-02	*Nectin2*	0,733
1,16E-02	*Cfl2*	0,733
2,75E-02	*Wfdc2*	0,734
4,39E-02	*Ifitm3*	0,736
2,78E-02	*Pdlim3*	0,738
2,52E-02	*Slc35e4*	0,739
1,35E-02	*Atox1*	0,740
3,20E-02	*Mfhas1*	0,740
6,32E-03	*Lrrc59*	0,741
1,50E-02	*Sh3gl1*	0,741
**1,27E-02**	***Plekhg6***	**0,742**
3,07E-02	*Ndufs7*	0,742
9,05E-03	*Src*	0,742
3,84E-02	*Krt19*	0,743
1,50E-02	*Git1*	0,744
9,12E-04	*Gm11361*	0,744
2,79E-02	*Rcc2*	0,744
4,69E-02	*Tmsb10*	0,745
2,75E-02	*Sgta*	0,746

3,72E-02	*Dnajc4*	0,749
1,57E-04	*Itpr3*	0,750
1,75E-03	*Gm38431*	0,750
4,07E-03	*Irf2bpl*	0,750
1,04E-02	*Gm43796*	0,750
2,82E-02	*Trappc6a*	0,751
1,20E-04	*Kdelr2*	0,751
1,45E-02	*Ndufa11*	0,753
2,48E-02	*Col18a1*	0,754
2,41E-02	*Stk26*	0,754
4,14E-03	*Osgin1*	0,755
4,64E-02	*Fam162a*	0,759
1,51E-02	*Fam25c*	0,761
3,37E-02	*Sgpl1*	0,761
1,95E-02	*Lmo4*	0,763
7,26E-03	*Ran*	0,764
9,22E-03	*Nacc1*	0,766
4,84E-02	*Gm20458*	0,766
1,72E-02	*Dvl3*	0,767
4,36E-03	*Rab5c*	0,767
2,84E-02	*Cmip*	0,767
3,85E-02	*Pkm*	0,768
4,49E-02	*Kdelr3*	0,769
3,43E-04	*Cope*	0,770
1,12E-02	*Irf1*	0,770
4,49E-02	*Arhgdia*	0,772
1,86E-02	*Mbd3*	0,774
4,15E-02	*Dda1*	0,776
1,35E-02	*C77080*	0,777
1,09E-02	*Stab1*	0,777
4,35E-02	*Sfn*	0,779
1,59E-02	*Mrpl54*	0,781
4,66E-02	*Ly6d*	0,782
3,79E-02	*Rps29*	0,782
2,32E-02	*Sdc1*	0,783
3,32E-02	*Lad1*	0,785
5,69E-03	*Traf2*	0,791
4,69E-02	*Prdx5*	0,793
2,48E-02	*Prrc2a*	0,794
3,79E-02	*Cltb*	0,799
2,73E-02	*Mtpn*	0,803
2,33E-02	*Pgls*	0,805
4,84E-02	*Szrd1*	0,809
3,58E-02	*Gltp*	0,810
2,95E-02	*U2af2*	0,812
2,75E-02	*Hoxa10*	0,814
1,28E-02	*Ndufa13*	0,816
3,44E-02	*Txndc5*	0,826

4,45E-02	*Serf2*	0,829
1,90E-02	*H2-D1*	0,841
4,80E-02	*B2m*	0,845
4,44E-02	*Arf1*	0,857

Upregulated genes in bladder		
padj	Gene	(FC) KO
4,85E-03	*Apobec2*	126,76
3,65E-02	*Ankrd2*	47,10
5,21E-05	*Ms4a4b*	34,24
1,81E-02	*Gapdh*	30,58
6,64E-03	*Mylk2*	21,89
1,35E-15	*Kcnc1*	18,26
7,14E-11	*Ms4a8a*	17,33
9,68E-08	*Pgpep1l*	17,09
5,60E-07	*Vxn*	15,66
4,68E-02	*Asb11*	13,23
4,33E-02	*Klhl40*	12,17
2,56E-02	*3425401B19Rik*	11,85
2,78E-02	*Hspb3*	11,60
**1,75E-02**	***Cmya5***	**11,16**
4,80E-03	*Myadml2*	10,73
5,47E-04	*Pik3c2g*	10,52
1,71E-03	*Alpk3*	9,84
3,17E-02	*Asb12*	9,79
1,08E-02	*Mrln*	9,76
1,82E-03	*Jsrp1*	9,47
2,22E-02	*Abcb4*	8,78
4,93E-03	*Cdh15*	8,68
8,79E-03	*Txlnb*	8,53
2,84E-02	*Asb15*	7,81
**5,75E-03**	***Xlr3a***	**7,63**
1,65E-05	*Plcd4*	7,07
4,80E-02	*Art5*	7,04
9,78E-03	*Tmem52*	6,77
1,30E-02	*Neu2*	6,46
2,86E-15	*Xlr3b*	6,11
**4,47E-04**	***Acan***	**6,07**
4,84E-02	*Mlip*	6,06
7,83E-03	*Phkg1*	5,98
7,71E-05	*Xlr4b*	5,79
3,44E-02	*Sec14l5*	5,78
1,55E-07	*Lrrc14b*	5,78
7,30E-03	*Hhatl*	5,22
**1,91E-02**	***Epyc***	**4,91**
3,66E-03	*Adprhl1*	4,70
4,45E-02	*Ip6k3*	4,64
1,29E-03	*Xirp1*	4,58
6,48E-04	*Ces1g*	4,43
**2,21E-09**	***Ftl1-ps1***	**4,39**
8,21E-03	*Asb16*	4,33
8,34E-03	*Fgl1*	4,18

Upregulated genes in colon		
padj	Gene	(FC) KO
3,19E-99	*Saa1*	37840,44
2,40E-06	*Saa2*	202,78
1,50E-03	*Xirp2*	120,05
**3,58E-82**	***Htatip2***	**94,74**
3,86E-02	*2200002J24Rik*	53,55
6,67E-03	*Gm14434*	31,64
8,85E-03	*Ifi206*	21,03
2,28E-03	*Tnfsf18*	20,45
8,68E-04	*Slurp1*	16,21
2,70E-03	*Wfdc12*	15,79
**5,80E-03**	***Acan***	**15,23**
1,43E-10	*Adamtsl2*	10,30
1,18E-06	*Fosl1*	9,08
**1,73E-05**	***Epyc***	**9,03**
1,35E-03	*Sln*	8,40
3,60E-02	*Mmp7*	8,39
1,08E-02	*Bhmt2*	8,09
3,88E-02	*Tnmd*	8,00
1,60E-04	*Trim29*	7,44
1,56E-05	*Krt84*	7,14
6,46E-05	*Ltbp2*	6,98
1,54E-18	*2010109I03Rik*	6,88
**2,52E-03**	***Lyz1***	**6,77**
3,34E-02	*Sele*	6,67
3,06E-02	*Tcap*	6,61
1,75E-02	*Serpina3m*	6,45
**1,32E-08**	***Sgcg***	**6,38**
4,61E-04	*Dio2*	5,53
4,60E-05	*Serpina3f*	5,44
1,89E-10	*Krt36*	5,41
8,74E-05	*Syt8*	5,21
2,21E-05	*Wisp2*	5,18
**3,30E-04**	***Itgbl1***	**5,08**
1,92E-05	*Plet1*	5,00
5,24E-03	*Bdkrb1*	4,68
5,75E-06	*Isx*	4,45
5,51E-05	*Lama1*	4,44
3,45E-04	*Aldh1a3*	4,26
7,82E-03	*Serpina3n*	4,25
4,10E-03	*Alpi*	4,14
1,33E-03	*Ido1*	4,13
1,38E-16	*Col23a1*	4,11
4,18E-05	*Gpbar1*	4,03
5,27E-04	*C2cd4b*	4,02
4,44E-02	*Gzmb*	3,88

3,58E-03	*Pla2g4e*	4,15
4,22E-02	*Serpinb6c*	4,14
2,90E-02	*Cpt1b*	3,99
2,22E-02	*Lrrc39*	3,97
1,35E-15	*Itih4*	3,97
9,78E-06	*Trdn*	3,78
**1,10E-25**	***Sgcg***	**3,72**
1,05E-03	*Ddit4l*	3,63
3,38E-02	*Cyp2e1*	3,49
7,10E-13	*Galnt9*	3,48
2,44E-08	*Uevld*	3,47
9,53E-04	*Usp2*	3,34
**2,27E-09**	***Mmp3***	**3,25**
1,42E-02	*Trpc6*	3,21
3,31E-07	*C6*	3,20
6,69E-03	*Grik4*	3,16
5,96E-10	*Rtn2*	3,15
1,56E-02	*Rgs22*	3,06
1,92E-02	*Wfdc6a*	3,04
1,00E-02	*Klhl33*	2,95
1,93E-02	*Cldn22*	2,91
1,02E-02	*Serpina3k*	2,88
2,77E-04	*Cyp1a1*	2,83
2,68E-02	*Ces1f*	2,83
7,26E-03	*Cstad*	2,80
**2,86E-15**	***Ftl1***	**2,79**
1,05E-02	*Angptl1*	2,75
7,46E-03	*Acsl6*	2,71
6,16E-03	*Klf15*	2,67
2,50E-21	*Ms4a4d*	2,66
4,92E-02	*Erfe*	2,65
4,36E-03	*Fn3k*	2,65
1,98E-02	*Myo18b*	2,58
**1,74E-16**	***Htatip2***	**2,58**
4,49E-02	*Cidea*	2,53
4,18E-05	*Tarsl2*	2,50
2,36E-02	*Itgb1bp2*	2,48
1,17E-02	*Mss51*	2,46
6,30E-03	*Fat3*	2,45
2,44E-04	*Strip2*	2,44
6,26E-03	*P2rx6*	2,42
**2,68E-06**	***Itgbl1***	**2,41**
9,52E-05	*Pfkm*	2,40
2,29E-02	*Srpk3*	2,38
**1,35E-02**	***Per1***	**2,38**
4,46E-02	*Usp13*	2,38
4,59E-07	*Trim36*	2,36
1,50E-05	*Klhl30*	2,34

1,26E-03	*Mmp10*	3,79
3,38E-03	*Wfdc13*	3,78
1,51E-13	*Serpine1*	3,73
6,31E-06	*Nt5c1a*	3,67
1,56E-04	*Scgb2b20*	3,59
2,91E-02	*Crlf1*	3,35
**7,95E-07**	***Cilp***	**3,35**
3,31E-03	*Ms4a10*	3,27
**1,50E-03**	***Ftl1-ps1***	**3,25**
**2,79E-05**	***Rrad***	**3,13**
1,74E-02	*Ggt1*	3,12
1,45E-03	*Ppy*	3,04
**9,55E-03**	***Xlr3a***	**3,03**
3,85E-02	*Klk1b11*	2,94
3,91E-05	*Apol7c*	2,91
**8,98E-03**	***Cmya5***	**2,90**
1,15E-03	*Kcnh1*	2,86
**4,67E-06**	***Nes***	**2,86**
2,92E-02	*Mefv*	2,79
9,55E-03	*Scgb2b7*	2,75
**6,39E-04**	***Mmp3***	**2,75**
8,74E-05	*Hbb-bs*	2,66
4,42E-02	*Gm14137*	2,66
4,68E-03	*Madcam1*	2,65
9,42E-05	*Apod*	2,65
3,78E-03	*Cd7*	2,63
5,36E-03	*Gm379*	2,58
9,06E-04	*Krt15*	2,56
6,24E-07	*BC025446*	2,52
1,77E-02	*Klk15*	2,50
2,28E-02	*Adamts4*	2,50
**8,74E-05**	***Ftl1***	**2,49**
2,14E-06	*Diras2*	2,49
1,94E-02	*Arc*	2,47
5,25E-05	*Cldn1*	2,47
3,48E-03	*Pla2g12b*	2,46
2,02E-02	*Plaur*	2,39
3,15E-02	*Slamf7*	2,37
2,08E-05	*Id1*	2,35
5,80E-03	*Epha2*	2,35
**5,04E-03**	***Fabp3***	**2,32**
1,67E-03	*Mzb1*	2,30
6,17E-04	*Fcgrt*	2,29
6,67E-03	*Dmp1*	2,25
2,48E-02	*Fmod*	2,23
3,84E-03	*Adamts1*	2,19
1,74E-02	*Areg*	2,18
**1,75E-05**	***Gem***	**2,17**

7,56E-03	*Lgals4*	2,34
2,10E-02	*Tspoap1*	2,32
3,09E-02	*Cox7a1*	2,31
**1,48E-02**	***Fabp3***	**2,31**
8,77E-04	*Tsc22d3*	2,29
2,94E-02	*Atp2b3*	2,24
3,55E-02	*Dpp6*	2,22
3,55E-02	*Ackr4*	2,22
1,06E-05	*Pcp4*	2,21
3,60E-02	*Tmod1*	2,20
4,91E-03	*Fam13a*	2,20
8,04E-03	*Macrod1*	2,16
4,59E-02	*Dkkl1*	2,14
3,30E-05	*Ttc23*	2,13
6,28E-04	*Lsamp*	2,12
8,19E-05	*Snx32*	2,10
3,73E-05	*Sult1a1*	2,09
**2,18E-05**	***Lyz1***	**2,08**
1,36E-03	*Tmem252*	2,06
6,43E-03	*9330182L06Rik*	2,06
5,06E-03	*Ramp1*	2,05
2,05E-03	*Kit*	2,05
1,19E-02	*Sv2b*	2,05
5,67E-04	*Fan1*	2,04
4,46E-04	*Olfr558*	2,04
2,70E-03	*Rbm24*	2,02
1,37E-06	*Mamdc2*	2,02
9,90E-08	*Galnt15*	2,01
1,62E-02	*Hlf*	2,01
1,20E-04	*Ccl8*	2,00
4,15E-02	*Rtl9*	1,99
4,74E-02	*Adssl1*	1,97
**1,99E-02**	***Gem***	**1,97**
4,43E-02	*Pxmp2*	1,96
8,80E-04	*Lmcd1*	1,96
7,53E-03	*Cacna2d3*	1,95
3,04E-03	*Ptgfr*	1,94
**6,39E-04**	***Nes***	**1,94**
3,03E-05	*Tmem38a*	1,91
3,08E-02	*Col6a4*	1,89
1,49E-02	*S100b*	1,87
1,34E-04	*Igsf5*	1,86
1,58E-02	*9330159F19Rik*	1,86
3,40E-02	*Inmt*	1,86
**5,95E-03**	***Cilp***	**1,84**
1,19E-04	*Ccdc3*	1,84
1,82E-02	*Phka1*	1,84
2,84E-02	*Amy1*	1,84

1,49E-05	*Anxa8*	2,17
1,06E-02	*Junb*	2,15
3,02E-03	*2010003K11Rik*	2,13
5,12E-04	*Klk1*	2,12
2,11E-02	*Pde9a*	2,11
1,78E-02	*Ptk6*	2,11
1,78E-02	*Tifa*	2,11
4,09E-02	*Tspan11*	2,09
8,85E-03	*Rbp1*	2,07
3,32E-02	*Alox12*	2,06
2,62E-04	*Rspo3*	2,05
1,74E-08	*Tnc*	2,04
1,07E-02	*Sorl1*	2,04
7,29E-03	*Dgat2*	2,02
2,40E-02	*Egr1*	1,98
3,96E-02	*Tef*	1,96
4,36E-04	*Igfbp5*	1,94
1,67E-02	*2410002F23Rik*	1,94
7,46E-03	*Sfrp4*	1,93
7,74E-03	*Igfbp4*	1,92
2,63E-02	*Col18a1*	1,92
2,91E-02	*H2-DMb1*	1,90
**4,09E-02**	***Smoc1***	**1,90**
3,08E-02	*Serpine2*	1,88
4,08E-02	*Cyp2d9*	1,87
1,94E-03	*Krba1*	1,87
3,03E-02	*Spink4*	1,86
3,86E-02	*Slc2a12*	1,86
1,09E-02	*Spon1*	1,86
6,17E-05	*Tat*	1,85
4,07E-02	*Capn8*	1,84
1,26E-03	*Pim3*	1,83
1,26E-02	*Lamc2*	1,83
1,37E-03	*Penk*	1,81
4,86E-02	*Thbs1*	1,79
4,86E-02	*Ccdc71l*	1,79
**3,59E-03**	***Per1***	**1,78**
9,87E-04	*Olfml3*	1,77
2,50E-02	*Gpx7*	1,77
**1,69E-02**	***Ctla2a***	**1,74**
7,82E-03	*Hbegf*	1,74
3,55E-02	*Lfng*	1,72
4,10E-03	*Rrp15*	1,70
1,13E-02	*Cxcl16*	1,69
3,23E-02	*Zdhhc14*	1,69
1,41E-02	*Ptrhd1*	1,68
2,95E-02	*Tmem86a*	1,67
1,41E-02	*Rgs2*	1,67

2,82E-02	*Slitrk6*	1,84
1,78E-04	*Tcea3*	1,83
1,34E-02	*Susd5*	1,82
3,67E-02	*Cep112*	1,82
2,38E-03	*Abca6*	1,82
2,42E-02	*Sgca*	1,81
6,49E-03	*Ak1*	1,81
1,32E-05	*Ssfa2*	1,80
2,20E-04	*Agl*	1,79
8,86E-06	*Fam13c*	1,78
6,10E-03	*Fam214a*	1,77
4,61E-02	*Clec4b1*	1,74
2,95E-02	*Spa17*	1,74
6,88E-03	*Mcee*	1,72
4,27E-02	*Unc45b*	1,70
3,73E-02	*Hoxc10*	1,68
3,73E-02	*Nov*	1,68
8,45E-05	*Ogfrl1*	1,67
1,58E-02	*C1qtnf3*	1,67
1,17E-03	*Hpse2*	1,67
2,98E-02	*Kcnq5*	1,66
**3,92E-02**	***Rrad***	**1,66**
2,69E-02	*Asph*	1,66
3,02E-02	*Ints6l*	1,64
2,70E-03	*Pdp2*	1,64
2,44E-06	*Tcp11l2*	1,64
1,33E-02	*Tex15*	1,64
1,39E-04	*Pcolce2*	1,63
4,64E-02	*Cdh6*	1,63
2,62E-04	*Thnsl2*	1,62
5,50E-04	*Abhd14b*	1,62
**4,14E-09**	***Fopnl***	**1,62**
1,06E-05	*Nudt19*	1,61
4,17E-03	*Ppp1r3c*	1,61
1,79E-02	*Nt5e*	1,60
4,79E-04	*Rgs7bp*	1,59
1,14E-02	*Cpxm2*	1,59
7,98E-03	*Gm44502*	1,58
1,33E-05	*Sspn*	1,58
5,57E-04	*Gm44503*	1,58
1,56E-03	*S1pr1*	1,58
3,34E-03	*Aox1*	1,58
3,40E-08	*Eif2a*	1,58
3,55E-02	*Rsph4a*	1,57
6,93E-03	*Sesn1*	1,57
3,27E-02	*Ifi207*	1,57
2,70E-03	*Ccpg1*	1,56
4,76E-05	*Zfp46*	1,56

3,23E-02	*Hspb7*	1,66
1,50E-02	*Kcnj8*	1,65
3,63E-02	*C1qa*	1,63
2,47E-02	*Ednra*	1,61
3,72E-02	*Coq7*	1,58
1,96E-02	*Gpx3*	1,55
4,94E-02	*Klf10*	1,54
**2,07E-02**	***Fopnl***	**1,54**
2,60E-02	*Bhlhe40*	1,52
**3,76E-02**	***Rps6***	**1,47**

2,32E-02	*Hist2h2be*	1,56
7,61E-04	*Atp1b2*	1,56
5,91E-03	*Fhod3*	1,56
2,84E-02	*Mfap4*	1,56
4,99E-03	*Fzd4*	1,56
2,41E-03	*Klhl7*	1,56
1,33E-02	*Epdr1*	1,55
3,73E-05	*Rps11*	1,55
2,93E-03	*Tpgs2*	1,55
4,20E-02	*Clip4*	1,55
4,23E-02	*Pdk2*	1,55
6,10E-03	*Ccrl2*	1,55
2,56E-03	*Vldlr*	1,55
1,68E-02	*Scara3*	1,54
4,08E-02	*L2hgdh*	1,53
2,78E-02	*Purg*	1,53
4,22E-03	*Rpl29*	1,52
1,75E-02	*Nqo1*	1,52
6,43E-06	*Cdon*	1,52
3,31E-03	*Eepd1*	1,52
8,02E-09	*C4b*	1,51
2,37E-03	*Thrb*	1,51
3,80E-03	*Ctsf*	1,51
3,86E-02	*Glul*	1,50
2,91E-02	*Xpa*	1,50
1,50E-02	*Vegfd*	1,50
7,33E-03	*Klf9*	1,50
1,26E-02	*Mturn*	1,49
4,23E-02	*Crebrf*	1,49
1,05E-02	*Cpe*	1,48
2,75E-02	*Fzd3*	1,48
3,58E-03	*Mtrf1*	1,48
**4,40E-02**	***Smoc1***	**1,47**
9,07E-05	*Eif3e*	1,47
3,66E-02	*Lrig1*	1,47
6,81E-03	*Gm49387*	1,47
3,65E-03	*Mill2*	1,47
4,58E-04	*Fxr1*	1,45
1,15E-03	*Tsc22d1*	1,45
3,51E-03	*Gstm5*	1,44
2,30E-02	*Myom1*	1,44
4,68E-02	*Wnt2b*	1,44
2,39E-03	*Hddc2*	1,44
1,76E-02	*Ppp1r1a*	1,44
7,50E-05	*Msrb1*	1,44
2,61E-03	*Stk38l*	1,43
2,37E-02	*Gyg*	1,43
4,02E-02	*Stard7*	1,43

2,74E-05	*Eif4b*	1,43
5,43E-03	*Polr3gl*	1,43
1,17E-02	*Erlec1*	1,43
8,45E-05	*Gtf2h1*	1,43
4,78E-02	*Tacc2*	1,43
3,03E-03	*Pdcd2*	1,42
4,40E-02	*Pcbd1*	1,42
3,84E-02	*Mpp6*	1,42
2,15E-03	*Gtf2e2*	1,42
1,81E-02	*Cbr4*	1,42
1,45E-02	*Acadsb*	1,41
2,95E-02	*Scn1b*	1,41
3,00E-03	*Rps27rt*	1,41
1,56E-02	*Jam3*	1,41
6,17E-03	*Atp2a2*	1,41
9,75E-04	*Pink1*	1,41
2,90E-02	*Arsg*	1,41
3,86E-03	*B230118H07Rik*	1,40
3,66E-03	*Fam8a1*	1,40
1,74E-02	*Fam69a*	1,40
8,65E-05	*Selenop*	1,39
4,92E-02	*Pnpla2*	1,39
1,27E-02	*Pla1a*	1,39
2,36E-02	*Gabra3*	1,39
1,41E-02	*B3galnt2*	1,39
4,59E-03	*Pigp*	1,38
1,68E-02	*Bambi*	1,38
2,75E-02	*Dpep1*	1,38
1,41E-03	*Hbp1*	1,38
4,68E-02	*Dnaja4*	1,38
3,54E-02	*Smpdl3a*	1,38
3,55E-02	*Fam210b*	1,38
1,95E-04	*Hspa8*	1,38
6,17E-03	*Sgcb*	1,38
5,75E-03	*Aaed1*	1,37
1,68E-02	*Jtb*	1,37
1,82E-02	*Tmem14a*	1,37
1,90E-05	*Cct4*	1,37
5,38E-05	*Eif3m*	1,37
1,72E-02	*Prmt9*	1,37
5,62E-03	*Igbp1*	1,37
1,68E-02	*Rai2*	1,37
3,70E-02	*43892*	1,36
6,30E-03	*Selenbp1*	1,36
4,46E-02	*Pigc*	1,36
**2,48E-02**	***Ctla2a***	**1,36**
4,40E-02	*Wrn*	1,36
2,08E-03	*Smim19*	1,36

1,11E-02	*Zranb1*	1,36
6,93E-03	*Mxi1*	1,35
2,40E-02	*Cyhr1*	1,35
1,16E-02	*Dnttip2*	1,35
5,95E-03	*Dcaf6*	1,35
3,89E-02	*Klhl8*	1,35
**1,25E-02**	***Rps6***	**1,35**
6,06E-05	*Mrpl9*	1,35
1,09E-02	*Mfap1a*	1,34
2,54E-04	*Nrd1*	1,34
3,38E-02	*Cyp4v3*	1,34
1,06E-02	*Saal1*	1,34
1,71E-02	*Rdh14*	1,34
1,27E-02	*Mmp2*	1,33
3,56E-02	*Ctps2*	1,33
3,73E-02	*Lrpprc*	1,33
2,61E-03	*Mrpl30*	1,33
3,77E-02	*Cdadc1*	1,33
4,35E-02	*Clasp1*	1,33
1,36E-03	*Rps27*	1,33
1,64E-02	*Zrsr1*	1,33
2,68E-02	*Lrrc20*	1,33
1,86E-03	*Igf2r*	1,32
8,65E-05	*Rtraf*	1,32
6,87E-04	*Dcaf8*	1,32
1,88E-03	*Nsmce4a*	1,32
3,97E-02	*Utp14a*	1,32
6,00E-05	*Rpl5*	1,32
4,02E-02	*Pgrmc2*	1,32
4,49E-03	*Ppa2*	1,32
1,93E-02	*Ktn1*	1,32
2,17E-03	*Dym*	1,31
2,26E-03	*Gab1*	1,31
2,12E-02	*Pmpcb*	1,31
6,61E-03	*Prrg3*	1,31
2,85E-03	*Commd3*	1,31
4,43E-02	*Ift74*	1,31
4,43E-02	*Pde5a*	1,31
3,47E-02	*Prepl*	1,31
1,79E-02	*Ttc3*	1,31
4,01E-02	*Jmy*	1,31
1,68E-02	*Rpa1*	1,31
3,70E-02	*Calcoco1*	1,31
1,05E-02	*Eci2*	1,30
3,96E-02	*Serf1*	1,30
1,80E-02	*Ndufb5*	1,30
7,17E-04	*Rps24*	1,30
3,09E-02	*Twnk*	1,30

4,40E-02	*Rhoq*	1,30
3,99E-02	*Etfrf1*	1,30
2,48E-02	*Bclaf1*	1,30
7,69E-03	*Glod4*	1,30
2,33E-02	*Akt3*	1,30
1,44E-02	*Tmem59*	1,30
2,24E-02	*Ndufa6*	1,30
2,22E-02	*Phyh*	1,30
3,31E-02	*Uchl5*	1,30
4,52E-02	*Dnajb6*	1,29
1,72E-03	*Rpl35a*	1,29
1,58E-02	*Tmem126b*	1,29
1,11E-03	*Nsa2*	1,29
2,35E-03	*Phax*	1,29
1,76E-02	*Zfp622*	1,29
3,82E-02	*AL731706.1*	1,28
2,22E-02	*Rpl39*	1,28
5,85E-04	*Npm1*	1,28
3,95E-02	*Cdk5rap3*	1,28
4,59E-02	*Mfn1*	1,28
1,19E-02	*Sugt1*	1,28
2,84E-02	*Ephx1*	1,28
2,90E-02	*Cds1*	1,28
7,35E-04	*Rps4x*	1,28
7,46E-03	*Dirc2*	1,28
3,77E-02	*Mia2*	1,28
2,67E-03	*Pfdn5*	1,28
5,75E-03	*Nol7*	1,28
2,48E-02	*Pnpla8*	1,28
1,99E-02	*Zfp869*	1,28
2,23E-02	*Oaz2*	1,28
1,01E-02	*Cd302*	1,27
5,65E-03	*Rpl7*	1,27
6,45E-03	*Rpl36a*	1,27
9,21E-03	*Eif2s3y*	1,27
4,61E-02	*Frg1*	1,27
7,50E-03	*Ak3*	1,27
7,34E-03	*Gm49356*	1,27
2,52E-02	*Pacs1*	1,26
2,75E-02	*Atraid*	1,26
2,29E-02	*Faf1*	1,26
4,15E-02	*Ankra2*	1,26
2,29E-02	*Paip1*	1,26
1,95E-02	*Sdf4*	1,26
4,39E-02	*Hmox2*	1,26
1,44E-02	*Dpm1*	1,26
8,65E-03	*Syf2*	1,26
3,59E-02	*Akr1b10*	1,26

7,65E-03	*Snw1*	1,25
2,59E-02	*Slu7*	1,25
3,47E-02	*Bcl2l14*	1,25
4,10E-02	*Nampt*	1,25
9,50E-03	*Ncl*	1,25
4,37E-02	*Ppm1b*	1,25
1,21E-02	*Uqcrh*	1,24
3,50E-02	*Golgb1*	1,24
1,26E-02	*Pik3ca*	1,24
4,07E-03	*Tspyl1*	1,24
5,24E-03	*Atp1b3*	1,24
4,22E-02	*Mcts1*	1,24
2,36E-02	*Eif4a2*	1,23
1,55E-02	*Zc3h15*	1,23
2,59E-02	*Atp5c1*	1,23
2,75E-02	*Actr10*	1,23
4,68E-03	*Hdac3*	1,23
4,56E-02	*Trmt2b*	1,23
1,34E-02	*S100a13*	1,23
2,68E-02	*Eef1b2*	1,23
3,95E-02	*Ngrn*	1,23
1,81E-02	*Eef1a1*	1,23
1,49E-02	*Gdi2*	1,23
3,25E-02	*AI597479*	1,23
3,97E-02	*Eif2b5*	1,22
3,87E-02	*Adh5*	1,22
4,48E-02	*Nom1*	1,22
3,37E-02	*Snx5*	1,22
2,52E-02	*Rnase4*	1,22
3,73E-02	*Rnf146*	1,22
4,86E-02	*Skp1a*	1,22
2,23E-02	*Paip2*	1,21
2,58E-03	*Naca*	1,21
3,76E-02	*Rpl22*	1,21
3,70E-02	*Ndufb9*	1,21
4,35E-02	*Rtcb*	1,21
2,30E-02	*Uqcrc2*	1,21
2,48E-02	*Rps25*	1,21
3,81E-02	*BC004004*	1,21
3,87E-02	*Gab2*	1,20
4,80E-02	*Rpl23*	1,20
4,22E-02	*Sfrp1*	1,20
3,38E-02	*Ipo5*	1,20
4,34E-02	*Cwc15*	1,19
4,46E-02	*Ltbp1*	1,18
4,28E-02	*Psmb1*	1,18
4,15E-02	*Bag1*	1,18
3,75E-02	*Rpl4*	1,18

3,89E-02	*Psma2*	1,17
